# Projecting the Hydrologic Impacts of Climate Change on Montane Wetlands

**DOI:** 10.1371/journal.pone.0136385

**Published:** 2015-09-02

**Authors:** Se-Yeun Lee, Maureen E. Ryan, Alan F. Hamlet, Wendy J. Palen, Joshua J. Lawler, Meghan Halabisky

**Affiliations:** 1 Climate Impacts Group, University of Washington, Seattle, Washington, United States of America; 2 School of Environmental and Forest Sciences, University of Washington, Seattle, Washington, United States of America; 3 Earth to Ocean Research Group, Department of Biology, Simon Fraser University, Burnaby, Canada; 4 Department of Civil and Environmental Engineering and Earth Sciences, University of Notre Dame, Notre Dame, Indiana, United States of America; Duke University, UNITED STATES

## Abstract

Wetlands are globally important ecosystems that provide critical services for natural communities and human society. Montane wetland ecosystems are expected to be among the most sensitive to changing climate, as their persistence depends on factors directly influenced by climate (e.g. precipitation, snowpack, evaporation). Despite their importance and climate sensitivity, wetlands tend to be understudied due to a lack of tools and data relative to what is available for other ecosystem types. Here, we develop and demonstrate a new method for projecting climate-induced hydrologic changes in montane wetlands. Using observed wetland water levels and soil moisture simulated by the physically based Variable Infiltration Capacity (VIC) hydrologic model, we developed site-specific regression models relating soil moisture to observed wetland water levels to simulate the hydrologic behavior of four types of montane wetlands (ephemeral, intermediate, perennial, permanent wetlands) in the U. S. Pacific Northwest. The hybrid models captured observed wetland dynamics in many cases, though were less robust in others. We then used these models to a) hindcast historical wetland behavior in response to observed climate variability (1916–2010 or later) and classify wetland types, and b) project the impacts of climate change on montane wetlands using global climate model scenarios for the 2040s and 2080s (A1B emissions scenario). These future projections show that climate-induced changes to key driving variables (reduced snowpack, higher evapotranspiration, extended summer drought) will result in earlier and faster drawdown in Pacific Northwest montane wetlands, leading to systematic reductions in water levels, shortened wetland hydroperiods, and increased probability of drying. Intermediate hydroperiod wetlands are projected to experience the greatest changes. For the 2080s scenario, widespread conversion of intermediate wetlands to fast-drying ephemeral wetlands will likely reduce wetland habitat availability for many species.

## Introduction

Wetlands are universally recognized as a globally important ecosystem type that provides food and habitat to a wide range of aquatic and terrestrial species. Wetlands also influence local hydrologic processes (e.g. groundwater recharge, surface water storage, and filtration) and biogeochemical cycles (nutrient cycling, carbon sequestration, sediment and contaminant transport) [1–7]. Wetlands are widely considered to be among the most vulnerable ecosystems to climate change [1, 3–4, 8–9], particularly in montane and alpine areas that are already experiencing accelerated effects of climate change such as reduced snowpack [2–4, 10–11]. However, the vulnerability of montane wetlands to climate change has not been quantified due in part to a dearth of empirical data and methods for modeling wetland dynamics. Data constraints also exacerbate a broader impression that these diverse and dynamic ecosystems may be too complex to model because of limited ability to characterize the hydrologic details in specific locations.

Mountain wetlands remain far less impacted by anthropogenic disturbance in comparison to those in the lowlands where water withdrawal, land-use change, and direct destruction (e.g. draining or filling) have caused substantial losses of wetlands over the past two centuries (e.g. >50% loss in the contiguous U.S.) [12]. For this reason, wetland data from alpine regions are an important resource for understanding baseline patterns and processes of hydrologic variation over time as a function of climate. Also, as climate change accelerates, impacts on montane and alpine areas are expected to intensify [3–5]. In the western U.S. where snow is an important component of the hydrologic cycle [13–16], increased warming in all seasons and projected decreased summer precipitation are likely to result in loss of mountain snowpack, increased evapotranspiration and increased soil-moisture stress in late summer [17–25]. We hypothesize that these hydrologic impacts would also result in loss of sensitive montane wetlands and systematic, landscape-scale changes in wetland structure and function in areas that are otherwise highly protected, such as National Parks and Wilderness Areas. These changes compound lower elevation losses of wetlands due to human development [12].

Hydrologic changes and potential shifts in the distribution of wetlands across montane landscapes could have substantial consequences for wetland species, ecosystems, and their extended biological networks. Because many wetland species sort along coarse hydrologic gradients, such as duration of inundation or wetland permanence, as a function of life history and developmental requirements [26–27], their vulnerability to climate-induced habitat loss will not be distributed evenly [28–29]. Therefore understanding climate impacts on different hydrologic classes of wetlands is critical to assessing vulnerability of particular species or types of wetland ecosystems, and more generally to understanding how the wide range of ecological services wetlands provide is likely to change over time.

The shortage of empirical data on wetland dynamics, coupled with the inherent complexities of studying wetlands (e.g. sheer number of sites, diverse range of wetland types, highly dynamic hydrologic responses, etc.), has allowed an untested assumption to persist, i.e. that wetlands are simply too complex to model with the limited site specific information and data that are available. The challenges are real: existing methods for wetland hydrologic modeling largely focus on individual sites and require a detailed understanding of fine-scale surface and subsurface hydrologic processes (e.g. [1, 30–31]). The data necessary for these models are almost universally lacking for wetlands in general and for small wetlands in particular, which are largely defined by the microtopography of their surroundings and connections to groundwater and small streams. Where data and models are available, a key limitation of such fine-scale approaches is that they generally cannot be applied to answer questions of how wetland dynamics will respond to climate change at the landscape scale. The limited approaches that do exist for predicting landscape-level response require longer time series of hydrometeorological data than are generally available (e.g. WETLANDSCAPE models) [1, 32].

Here we present new methods not only for reconstructing historical dynamics when observed hydrologic data are limited, thereby filling important data gaps, but also for projecting the impacts of future climate change on wetland water levels. We draw upon an empirical dataset (125 ponds across 5 National Parks and Wilderness Areas in the U.S. Pacific Northwest) to develop a new set of hydrologic models for four classes of montane wetlands that vary in hydroperiod. Our methods generate projections at two spatial scales: a) individual wetlands and b) landscape scales. The approaches we develop allow us to quantify the combined effects of projected future reductions in snowpack and of warmer and drier summers on mountain wetlands. Looking forward, these projections can be used to develop testable hypotheses for wetlands research, develop climate adaptation strategies, and provide specific decision support for managers tasked with wetlands conservation, restoration, and management.

## Materials and Methods

### Wetland classification

We define wetlands broadly as any area where shallow surface water collects. Wetlands exist on a hydrologic continuum and many different methods of wetland classification exist (e.g. Cowardin, hydrogeomorphic (HGM)). We use a simple hydrologic classification scheme based on hydroperiod and wetland sensitivity to climate variability to characterize four ecologically relevant types of wetlands–ephemeral, intermediate, perennial, and permanent (Table 1). This approach is similar to other hydrologic classifications based on hydroperiod [33] and volumetric water loss [34] in that it relates inherently continuous hydrologic variation in water permanence and periodicity to more discrete ecological thresholds determined by species’ development and life history requirements (e.g. number of months or years that ponds must hold water for an insect, frog, or salamander to develop and metamorphose).

**Table 1:** Hydrologic Classification of Montane Wetland Types.

Wetland Classification	Hydrologic Characteristics	Ecosystem Characteristics
**Ephemeral**	Ephemeral or short-hydroperiod wetlands dry in most years, in some cases soon after the cessation of snowmelt or seasonal rains.	Ephemeral montane wetlands are not used by many animals due to their extremely brief inundation, but may support wetland plants.
**Intermediate**	Intermediate-hydroperiod wetlands tend to dry in late summer or early fall in years with low precipitation. During relatively wet years, they hold water year-round. Water levels fluctuate considerably during the summer.	Intermediate-hydroperiod wetlands support populations of fast-developing amphibians, invertebrates with resting egg stages that can survive desiccation, migratory birds, mesopredators, and wetland-obligate plants.
**Perennial**	Perennial wetlands do not dry except in the most extreme dry years, but often lose a substantial percentage of their volume during dry periods.	Perennial wetlands often support the greatest diversity and abundance of amphibians and invertebrates, including fast-developing species and those that require multiple years to complete larval development in high elevation environments, while lacking predators such as introduced fish that often reduce species diversity (Bahls 1992). Wetland-obligate plants, birds, and mesopredators, may also rely on perennial wetlands.
**Permanent**	Permanent wetlands do not dry and lose a relatively small percentage of their volume even during unusually dry periods.	Permanent wetlands support a broad range of mammals and birds, and can be used by the full suite of wetland-breeding amphibians and most invertebrates, though increased predation (by native and introduced predators) limits actual occupancy. Primary poductivity depends in part on the amount of shallow littoral habitat. Wetland macrophytes are common.

Quantitatively we define the four wetland types based on average minimum water levels. Generally shallow **ephemeral wetlands** dry completely or drop, on average, below 3% of their maximum water levels. Ephemeral wetlands are functionally dry for most aquatic species in most years, but may have water for the entire summer in unusually wet periods. **Intermediate wetlands** drop to between 3% and 33% of their maximum water levels on average and may dry completely in some years. **Perennial wetlands** drop to a mean of 33% to 70% of their maximum water levels and only in extreme droughts do they dry completely. For **permanent wetlands** (lakes and large ponds), the average minimum water levels generally remain greater than 70% of their maximum water levels and are never dry in the historical record. These four types represent general hydrologic classes of wetlands that can be related to more detailed hydrogeomorphic wetland-classification systems such as those used in U.S. Natural Heritage programs and for conservation decision making [35–36].

### Observed wetland data

**Fig 1:**
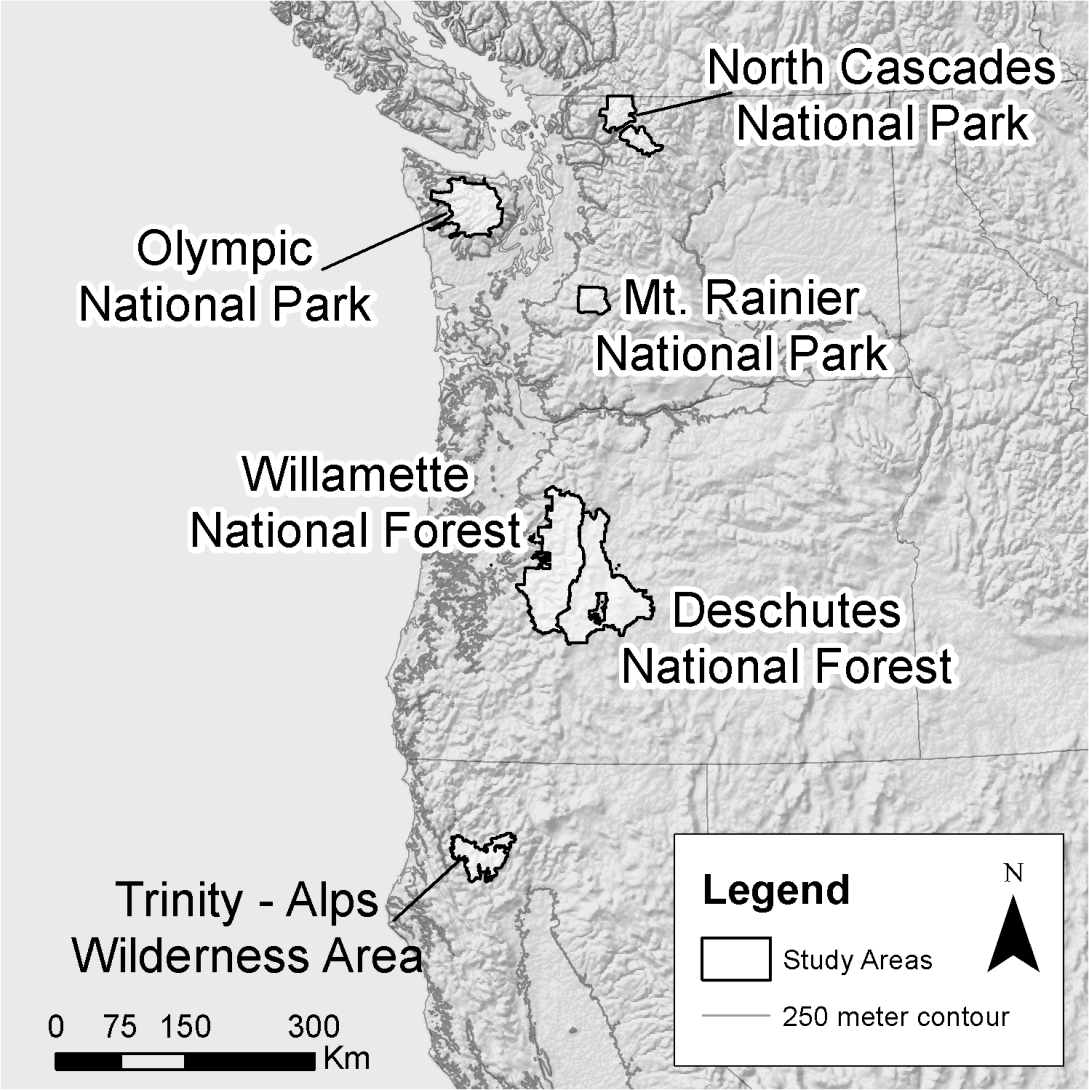
Regions with observed wetland data used to calibrate montane wetland models.

**Fig 2:**
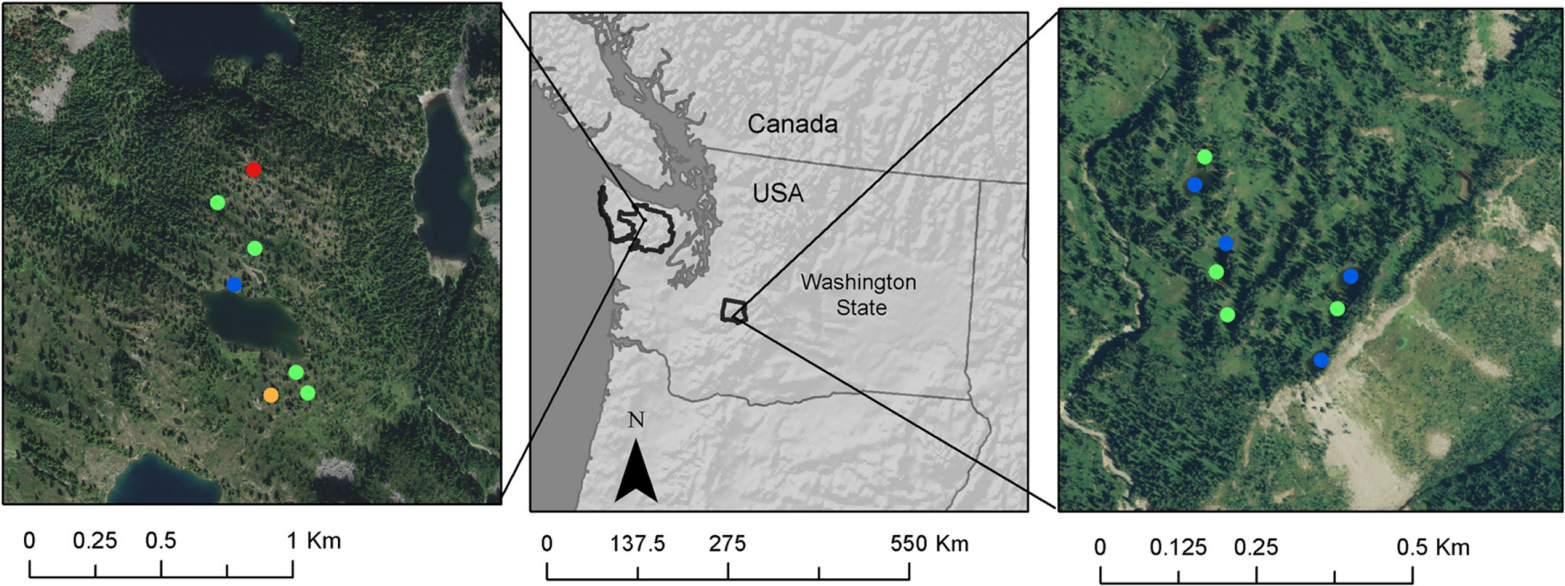
Locations of focal field sites in Seven Lakes Basin in Olympic National Park (left) and Mazama Ridge/High Lakes in Mount Rainier National Park (right) with multiple years of hydrologic data. Points indicate the pond type: red is ephemeral, orange is intermediate, green is perennial, and blue is permanent.

We collected detailed data on wetland hydrology (wetland depths and spatial extent) for 121 montane wetlands through in situ physical monitoring (3–6 site visits over the course of the summer and fall of 2012) in Olympic National Park, Mount Rainier National Park, and North Cascades National Park (Tables 2 and S1). Field permits for hydrologic monitoring were granted by the National Park Service (Olympic, Mount Rainier, and North Cascades National Parks). In a subset of wetlands, we also estimated wetland depths using iButton temperature dataloggers. To do so, we installed iButtons along transects from the edge of the wetland to the deepest accessible point in the wetland. We identified the date at which wetland water levels dropped below each iButton based on changes in the variance in temperature (measured every two hours). Because air temperatures fluctuate more dramatically than water temperatures, it is possible to compare temperatures of iButtons along each transect to iButtons placed in the open air adjacent to the wetland to determine when the iButton was submerged or exposed to the air. Our physical depth measurements validated the estimates of water level derived from the iButton transects. This larger 2012 dataset supplemented several smaller historical datasets that we also used in building the models. These included 1) measured wetland water depths for 7 montane wetlands in Olympic National Park from the summer of 2000, 2) wetland water volume estimates for 10 montane wetlands in Mount Rainier National Park from June through September 1992 [34], and 3) multiple years of observed wetland depth data for one intermediate and two perennial wetlands in Oregon [37] and one intermediate wetland in California [38–39] (Figs 1 and 2 and Tables 2 and S1). In total we used data from 125 wetlands.

**Table 2:** Summary of observed wetland data used in the study.

	Type	Period	Number of Wetlands				Data Source
			Ephemeral	Intermediate	Perennial	Permanent	
Mt. Rainier	Vol.	Year 1992	–	–	5	5	Girdner & Larson (1995)
National Park, WA	Depth	Year 2012	4	9	4	13	Field Measurement, M. Ryan
	Depth	Year 2012	2	–	2	1	iButton, M. Ryan
Olympic	Depth	Year 2000	1	–	4	1	Field data, W. Palen
National Park, WA	Depth	Year 2012	17	19	20	21	Field Measurement, M. Ryan
	Depth	Year 2012	1	–	4	–	iButton, M. Ryan
North Cascades	Depth	Year 2012	2	–	–	1	Field Measurement, M. Ryan
National Park, WA	Depth	Year 2012	–	–	–	1	iButton, M. Ryan
Willamette National Forest, OR	Depth	Years 2003–2006	–	1	1	–	Field Measurement, C. Pearl
Deschutes National Forest, OR	Depth	Year 2003 & 2006	–	–	1	–	Field Measurement, C. Pearl
Trinity Alps Wilderness, CA	Depth	Years 2003–2007	–	1	–	–	Field Measurement, J. Garwood

### Macro-scale hydrologic model

**Fig 3:**
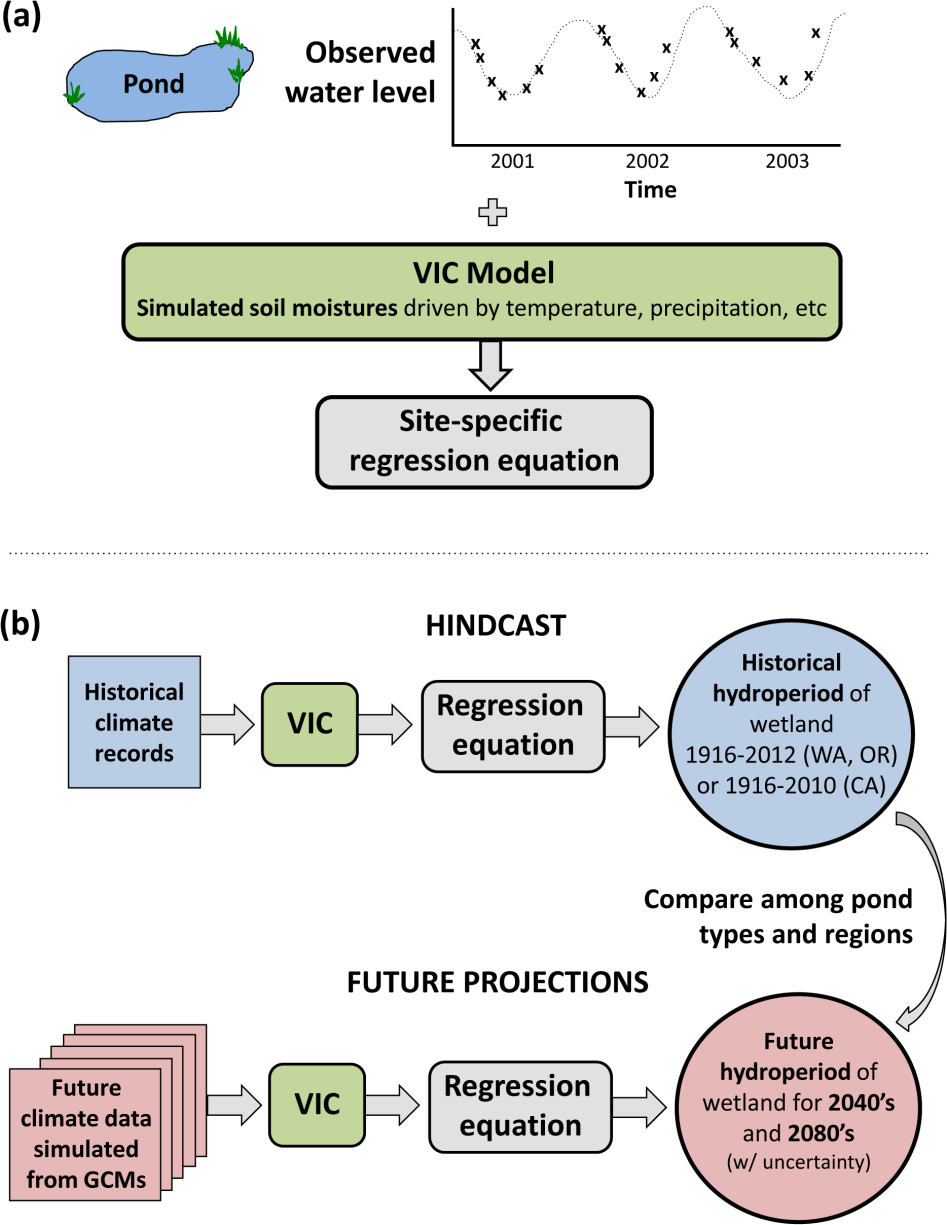
Schematic diagram of the method of projecting and hindcasting wetland hydrology using the VIC hydrologic model calibrated with empirical data and driven by historical or simulated future climate inputs.

To hindcast over a large number of retrospective years and to project climate-induced hydrologic change, we used the macro-scale Variable Infiltration Capacity (VIC) hydrologic model [40–41], implemented at 1/16^th^ degree resolution (roughly 5 km by 7 km) over the Pacific Northwest (PNW) and California (CA). The PNW model implementation, historical simulations, and climate change scenarios are described in detail by Elsner et al. (2010), Hamlet et al. (2013), and Tohver et al. (2014) [17–18, 42]. The VIC model implementation over the western U.S. is described by Salathé et al. (2013) [43]. For given input data including temperature, precipitation, wind, vapor pressure, net incoming longwave and shortwave radiation, and air pressure, the VIC hydrological model simulates daily water balance variables such as snowpack, evapotranspiration, runoff, baseflow and soil moisture in three soil layers comprising the first several meters of soil. Simulations at each VIC grid cell cover an extended historical time period (water-years (i.e. October 1 to September 30) 1916–2012 for the PNW and 1916–2010 for CA). We used these water balance variables to develop empirical regression models of wetland response as described below and to hindcast historical variability of water levels in wetlands over 97 (PNW) and 95 years (CA) (Fig 3).

### Regression models of wetland water levels

Wetlands even in close proximity to each other can show very different hydrologic behavior reflecting different pond types and different environments surrounding each wetland. Thus we first considered each unique wetland, investigated the best predictor per wetland, and used the best predictor to construct a single regression model as discussed below. This approach allows us to observe which predictors tend to offer the best fit across wetlands and wetland types, enabling us to estimate the impacts of climate change at the landscape scale.

As a first step, we used the correlation coefficient (Pearson’s R) to identify the strongest relationships between observed wetland water level at each site and a suite of daily water balance variables that were input to, or output from, the VIC models, including precipitation, evaporation, runoff, snow-water equivalent, and simulated soil moisture in the three different layers. Secondly, we constructed empirical models of wetland response by fitting regression equations to observed wetland water levels (volumes or depths) using the best-correlated water balance variable. We used the R (version 3.2.0) packages “stats” and “hydroGOF” to develop regression models and generate model statistics [44–45]. Because montane wetlands are relatively undisturbed by human changes in land use, when making future projections we assumed that the fitted regression relationships between wetland response and the soil moisture of the best-correlated water balance variable will not change with time. We developed a regression model using only observed data during drawdown periods because we did not have enough observed data during the rapid refill seasons to develop robust regression models for these. Also, for this system, the ecological consequences of altered timing of wetland drawdown and drying are greater than shifting timing of wetland refill, so we focused on the drawdown cycle for this reason as well.

For most Mount Rainier, Olympic, and North Cascades sites, we had only a single year of data from which to fit the regression models. However, when multiple-years of observations were available (e.g. for some sites in Mount Rainier and Olympic National Park and for all sites in Oregon and California), we investigated the uncertainty in the projections when fitting to a single year of data. For Oregon and California sites that showed similar gradual drawdown patterns for observed years, we fitted a separate regression model for each individual year of multiple years of observations, developed simulations for all years using each of these models, and then calculated the squared correlation coefficient (R^2^) using all observations and the corresponding simulations for each model. We then used the single-year regression model showing the highest squared correlation coefficient (R^2^) for all the available data to simulate historical wetland response and the associated climate-change response. We used the regression models fitted to other years to estimate the uncertainty in the simulations due to uncertainty in the regression parameters for individual years. For the Willamette National Forest site, for example, the squared correlation coefficient (R^2^) for the three regression models using 2003, 2005 and 2006 data to train the regression models, was 0.49, 0.88 and 0.75, respectively. Therefore, we used the regression model fitted on the 2005 observed data to simulate the historical response and used the regression models for years 2003 and 2006 to estimate the uncertainty of the simulation due to uncertainty in the regression fit.

The Mount Rainier and Olympic National Park sites with two years of observed data presented somewhat different drawdown patterns between years in response to different weather conditions. For example, one site in ONP showed gradual drawdown for year 2012 but, intermittent drawdown and frequent partial refill events for 2000. Thus, we developed a regression model using the earlier year’s data (e.g. 2000 data for Olympic National Park and 1992 for Mount Rainier), which had a greater number of observations and showed more dynamic changes in water levels. We then used the 2012 data from both regions to validate the regression models in the context of potentially different patterns of drawdown and refill across years. We used root mean squared error (RMSE) to evaluate how the regression model developed using the earlier year’s data performed to simulate the 2012 data.

### Climate change scenarios

To simulate potential future changes in water dynamics of individual montane wetlands in the PNW, we used simulated climate from the ECHAM5 general circulation model (GCM) forced by the A1B emissions scenario, which approximates the average conditions simulated for the region by ten different GCMs forced by the same emissions scenario. Climate projections were downscaled with the Hybrid Delta (HD) method described in detail in Appendix A of Tohver et al. (2014) [42]. Briefly, the HD method uses quantile mapping techniques to produce the transformed monthly observed climate data (water-years 1916–2006; a 91-year time series) in response to 30 years of monthly GCM projections for two future time periods: the 2040s (2030–2059) and the 2080s (2070–2099). The future monthly values are then used to rescale the daily values from the observed month to produce a future daily time series. As a result, the HD approach provides 91 years of observed variability ("1916–2006") for each future time period, and is directly comparable to the historical record on an event basis from 1916–2006. Note that for historical runs we extended the VIC runs from 1916–2006 to 1916–2012 in order to use field data obtained in 2012. However, for climate scenarios we used existing data developed by Hamlet et al. (2013) [17] that have 91 years of observed variability, projected for future time periods. For landscape scale projections of the probability of drying for intermediate wetlands in Washington, we used an ensemble of ten climate change projections from 10 different GCMs forced by the A1B scenarios [17]. We report the average of these results.

For California, we used the ECHAM5 A1B scenario based on the Distributed Delta (DD) downscaling approach that is described by Littell et al. (2011) [46] and Salathé et al. (2013) [43]. Although, ideally, we would have used data downscaled with the same approach for the California sites as we did for the Oregon and Washington sites, these data were the ones readily available from previous studies. Similar to the HD approach, the DD scenarios construct a 95-year time series for two future time periods, which have the same number of years as the observations used in the downscaling. Although there are some differences in details between this downscaling method and the HD approach described above (e.g. length of record, method by which the changes in climate are applied to the historical time series), for our use here these differences are not particularly important. Both methods incorporate the spatial distribution of temperature and precipitation changes from GCMs, and changes in the central tendency of projected temperature and precipitation in each region are similar in each case.

### Historical reconstruction, future projections, and frequency analysis of wetland dynamics

We estimated the average minimum wetland water level for both the historical runs and two future scenarios with time series behavior derived from the historical period. The average minimum wetland water levels were used to classify wetland types and evaluate the impacts of climate change on minimum wetland water levels.

To project changes in the distribution and behavior of wetlands at landscape scales, we investigated a threshold for wetland drying (when ponds reach 0% of maximum water level) that can be applied generally across different locations and soil types. To do so, we evaluated the relationship between wetland drying and “field capacity.” Field capacity is the volumetric soil water content of soil that is partly saturated, but drains very slowly by gravity. This metric relates directly to our best predictors (soil moistures, as discussed below). We converted absolute soil moisture values generated by the VIC model to the field capacities that vary with soil type. We then compared simulated soil moisture with wetland water levels for intermediate wetlands at Mount Rainier and Olympic National Parks to find a threshold below which wetlands tend to dry out. Finally, we used the mean threshold of drying across regions and soil types as a proxy for intermediate wetland drying for each VIC grid cell in the landscape scale analysis.

To calculate the probability of drying in each cell we counted the number of water-years for which the magnitude of soil moisture in the bottom soil layer was less than the drying threshold, and then divided by the total number of water-years. Thus the probability of wetland drying ranges from 0 to 1, with a higher value indicating more frequent wetland drying. We calculated the probability of wetland drying for the historical run and for all ten Hybrid Delta A1B scenarios for the 2080s, averaging the latter results for presentation in the plots. We then estimated the change in the probability of wetland drying by subtracting the probability of wetland drying for the historical run from that for the average value for the 2080s, with a positive value indicating an increase in the probability of drying for the 2080s.

## Results

### Historical reconstruction of wetland dynamics

The soil moistures in the middle and bottom soil layers were better correlated with observed wetland water levels than other water balance variables (S1 Fig). The best predictor differed among years and regions. Soil moisture in the middle soil layer was the best predictor for most of wetlands based on observed data in 1992 for Mount Rainier National Park and 2000 for Olympic National Park (S2 Fig). However the strength of correlations differed substantially between the 1992 Mount Rainier and 2000 Olympics datasets, with fairly strong correlations between wetland water levels and middle layer soil moisture in Mount Rainier and far weaker correlations and poor overall model performance in the Olympic sites in 2000. Overall, though, soil moisture in the bottom soil layer was the best predictor for all wetlands except the 1992 Mount Rainier and 2000 Olympics datasets, with fairly robust correlations between simulated and empirical data (S2 Fig). After choosing the best single predictor for each wetland, we developed a regression model for each individual wetland.

**Fig 4:**
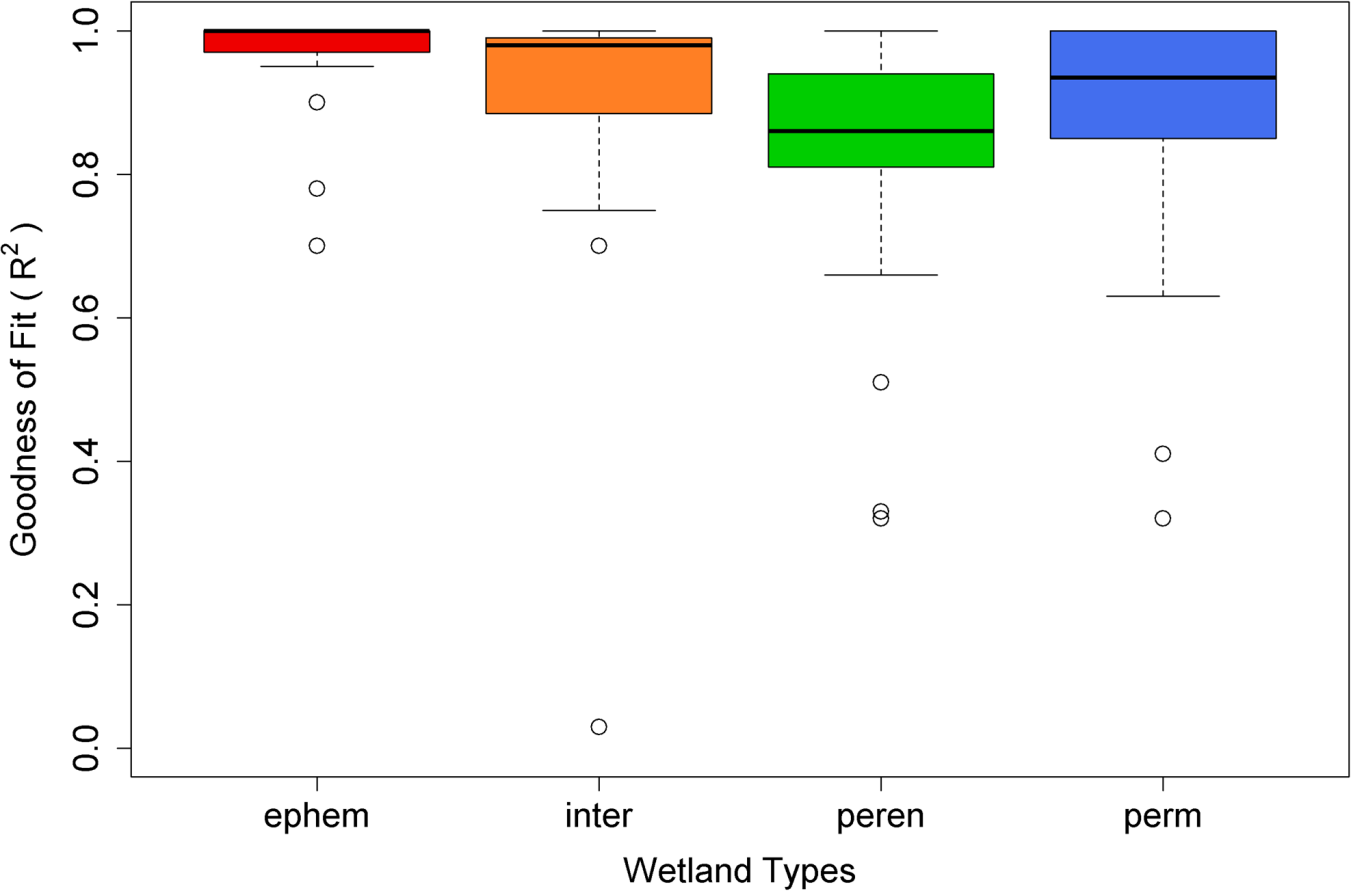
Boxplot of goodness of fit (R^2^) between observed and simulated data for each wetland type.

**Fig 5:**
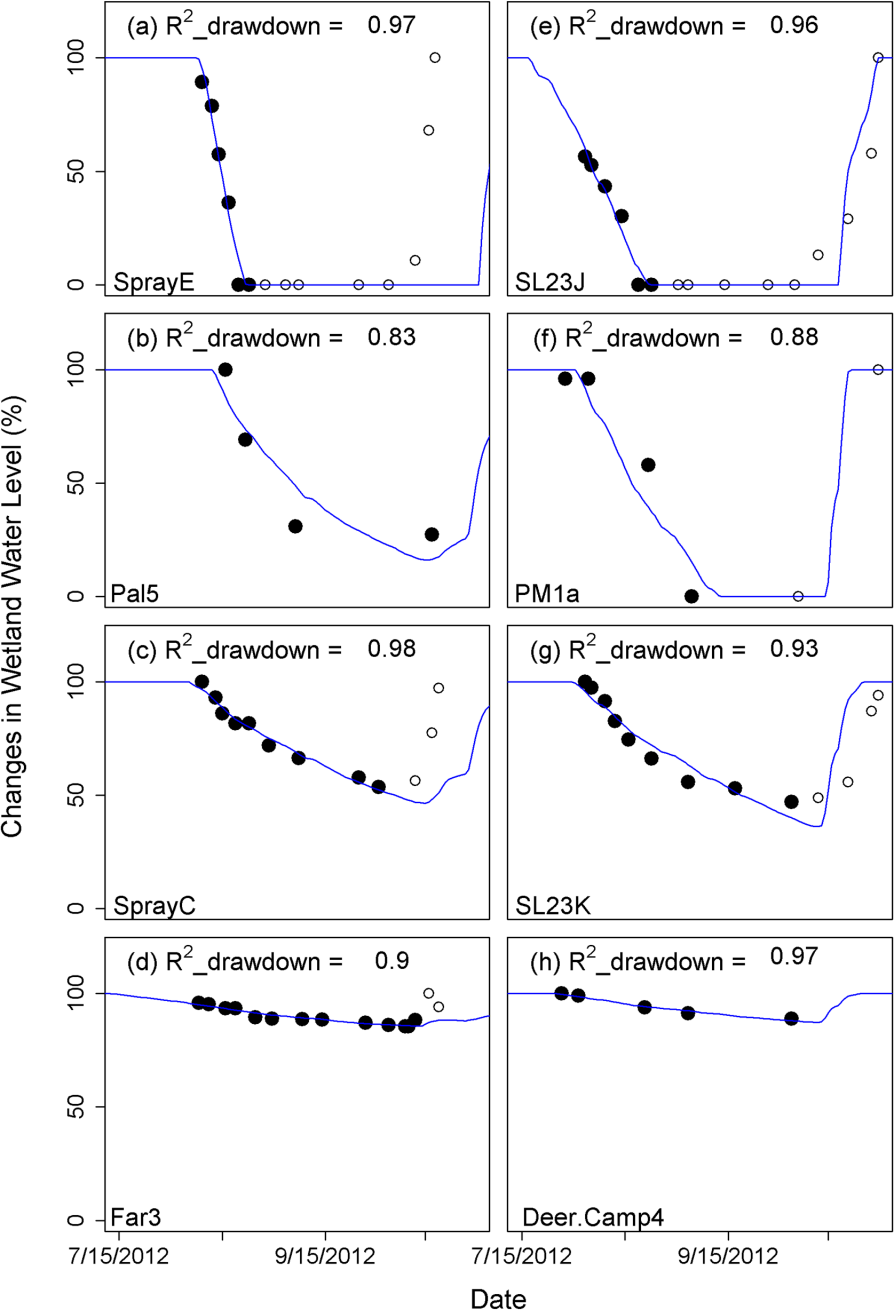
Four representative wetlands in Mt. Rainier National Park, WA (a-d) and in Olympic National Park, WA (e-h) for ephemeral hydroperiod (a,e), intermediate hydroperiod(b,f), perennial (c,g) and permanent wetland (d,h). Solid circles show observed data that are used for developing regression model and calculating R^2^ values and open circles are remaining observed data. Blue lines are simulated wetland levels.

**Fig 6:**
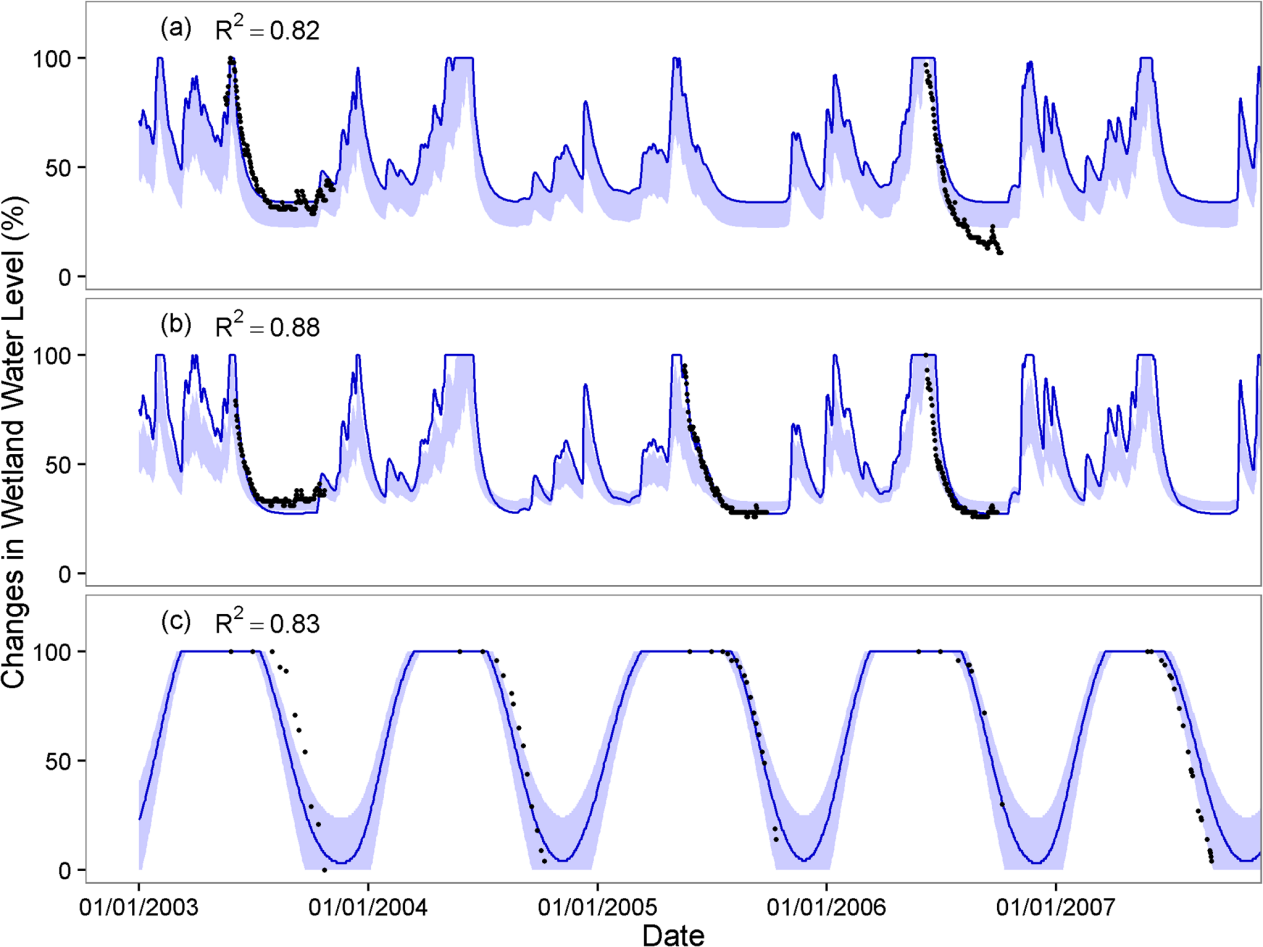
Observed wetland water levels compared with simulation using the regression models for a) Deschutes National Forest, OR, b) Willamette National Forest, OR, and c) Trinity Alps Wilderness, CA. Dark blue lines are produced by the regression equation deriving from the best fit from all available years. Blue bands show the range of uncertainty associated with alternate regression parameters deriving from other years of data.

The R^2^ values showed that the regression models match empirical observations reasonably well, with a mean R^2^ for all for wetland types above 0.8, though a few wetlands (6 out of 125; 4.8%) had an R^2^ value below 0.6 (Figs 4 and S3). Overall, ephemeral and intermediate wetlands showed the best model fits, while perennial ponds had the weakest fit among wetland types (Fig 4). The observed timing and pattern of seasonal drawdown generally were captured well by simulations for montane wetlands across the majority of our different sites (0.82 < R^2^ < 0.98) (Figs 5 and 6). Fig 5 illustrates representative wetlands for each pond type from Mount Rainier (left column) and Olympic National Parks (right column), selected from sites with the most observed data. Exceptions to the overall good model performance were the minimum water level for the year 2006 for Deschutes National Forest, Oregon (Fig 6A) and the timing of drawdown for the year 2003 in Trinity Alps Wilderness, California (Fig 6C). For the Deschutes National Forest, the observed minimum water level for year 2006 was 13% of full capacity but the simulation minimum was 22–34%. For the Trinity Alps Wilderness site, the wetland started drawdown about one month later in 2003 compared to the other years but the simulation was not able to reproduce this timing.

**Fig 7:**
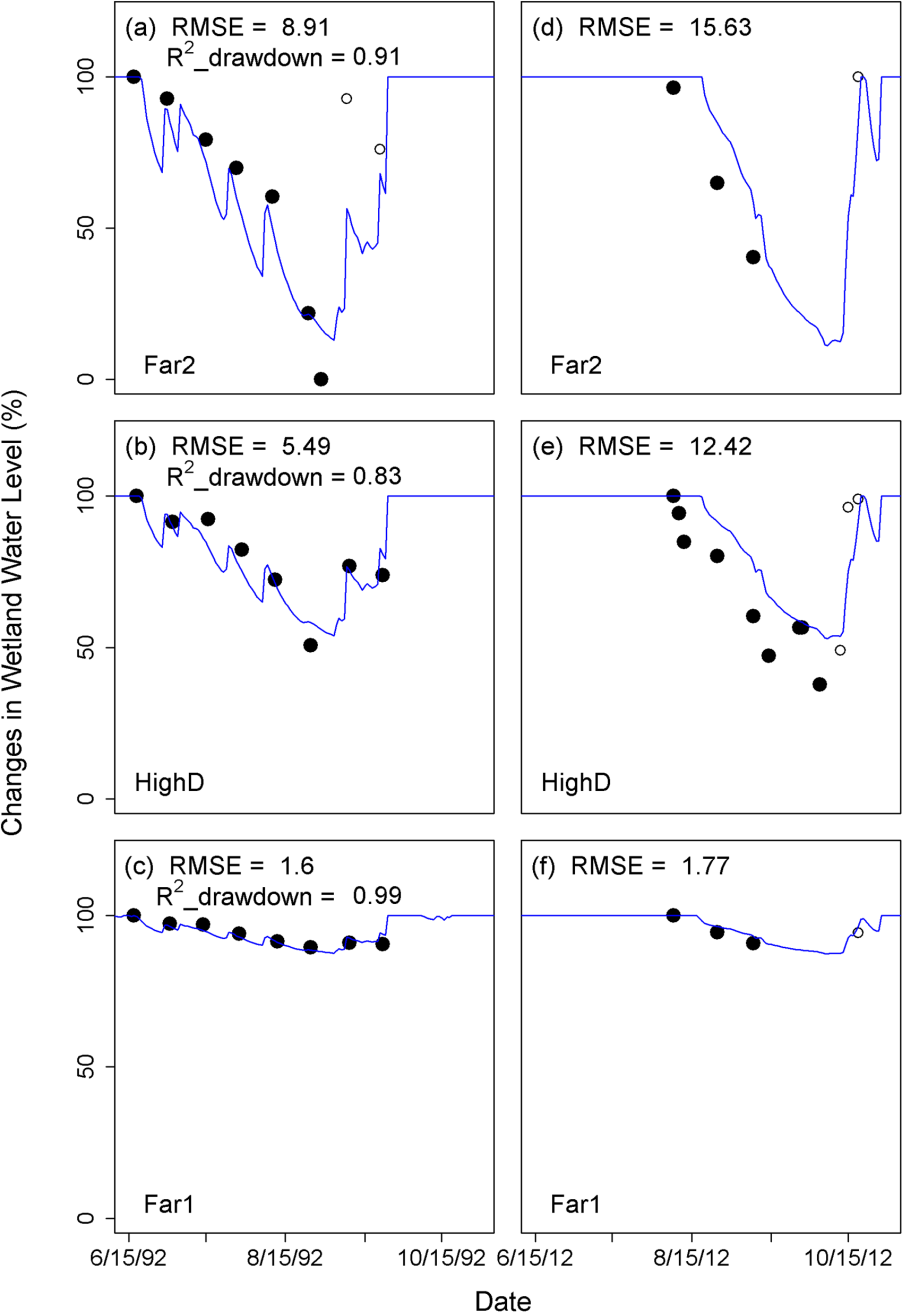
Observed wetland water level for year 1992 (a-c) and for year 2012 (d-f) for ponds at Mount Rainier National Park compared with the simulated wetland water levels using the regression model that were developed based on 1992 observation. Solid circles show observed data that are used for developing regression model and/or for calculating R^2^ values and open circles are the other observed data. RMSE is the root mean squared error, which allows comparison of model fit for the two datasets.

For sites where multiple years of data existed, we found considerable uncertainty and variation in model performance (Figs 6 and 7 and S4). For example, different regression models for the Trinity Alps (CA) site, constructed using different years of data, showed an average difference of 47 days between the earliest and latest prediction of minimum water levels in summer (Fig 6C). While the regression model for Mount Rainier National Park developed based on 1992 data (0.83 < R^2^ < 0.99; 1.60 < RMSE < 8.91) also captured the observed drawdown at the same wetlands in 2012 (1.77 < RMSE < 15.63) (Fig 7), model performance across different years was poor for Olympic National Park. For Olympic National Park, the regression model based on 2000 observations (0.51 < R^2^ < 0.81; 4.28 < RMSE < 19.67) missed the timing of drawdown and/or minimum water levels observed in 2012 (17.57 < RMSE < 49.12) (S4 Fig). We therefore might expect comparable levels of uncertainty in the performance of the single-year regression fits for the broader suite of wetlands at Mount Rainier, Olympic and North Cascades National Parks. These issues related to the performance of single-year regression fits once again highlight the need for improved monitoring of wetlands over multiple years.

In investigating the threshold for wetland drying we found that, on average across regions, wetland drying occurred when soil moisture was at roughly 100% field capacity, though there was considerable variation among sites (S5 Fig). Therefore to have a single metric for landscape-scale analysis, we use 100% field capacity as a mean threshold of drying and proxy for intermediate wetland drying for each VIC grid cell. These values help quantify systematic changes in wetland response over large areas in response to climate change, but probably do not represent the details of individual wetland responses very well.

### Future climate projections of wetland dynamics

**Fig 8:**
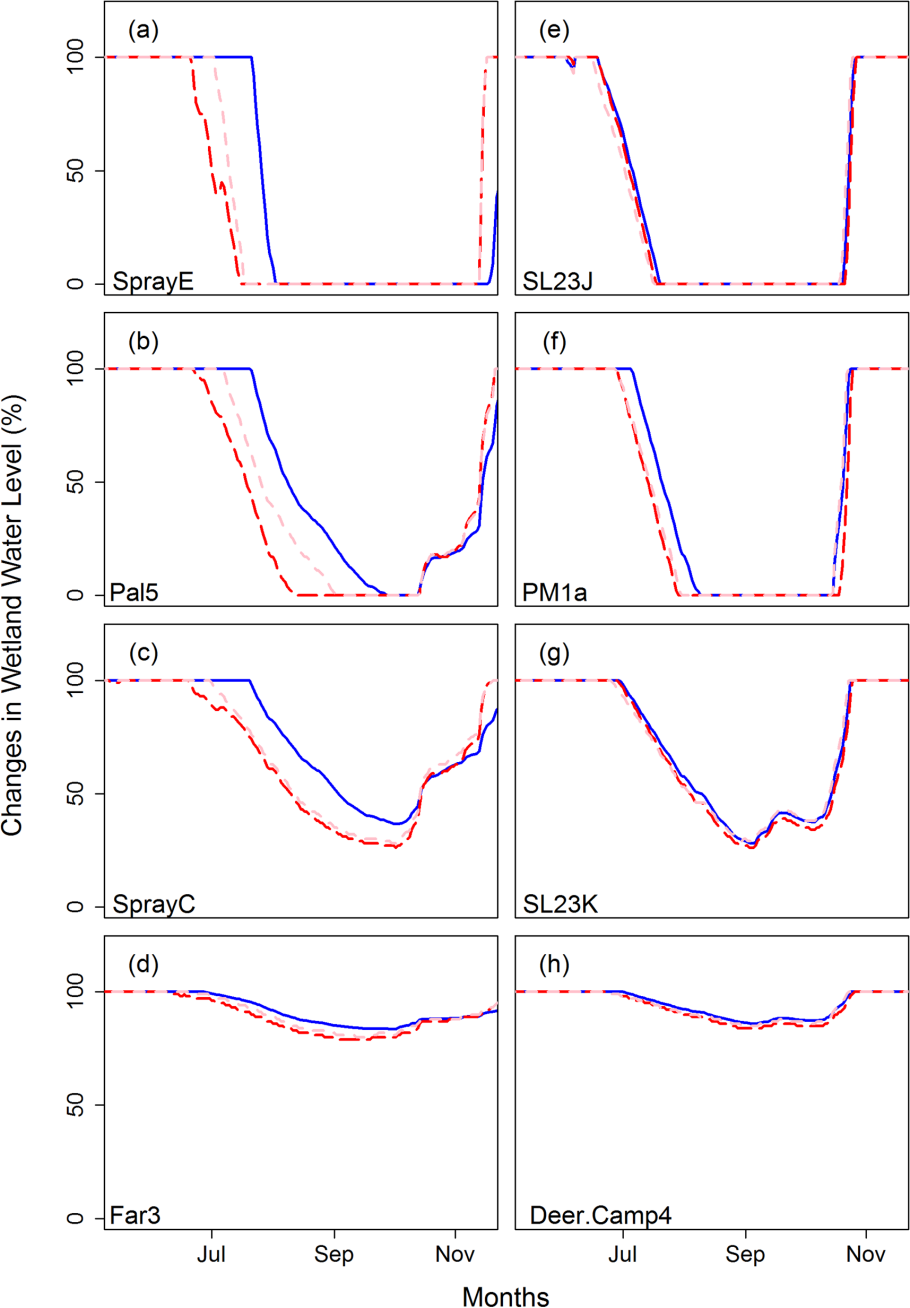
Projected wetland response to climate change for sites in Mt. Rainier National Park, WA (a-d) and in Olympic National Park, WA (e-h) for ephemeral hydroperiod (a,e), intermediate hydroperiod (b,f), perennial (c,g) and permanent wetlands (d,h). Blue solid lines are wetland hydrographs for representative years 1998 (a-d) and 1985 (e-h) and pink and read dashed lines show wetland hydrographs of year 1998 (a-d) and 1985 (e-h) with climate change perturbation for the 2040s and 2080s, respectively.

We found consistent projected effects of climate change on all classes of wetlands, including earlier drawdown, a more rapid recession rate in summer, and reduced minimum water levels (Figs 8 and S6). Overall, water levels in ephemeral or intermediate wetlands are most sensitive to climate change (Fig 8A, 8B, 8E and 8F). In ephemeral or intermediate wetlands, the effects of earlier drawdown, more rapid summer recession rate, and reduced minimum water levels result in a longer dry season in summer (Figs 8A, 8B, 8E and 8F and S6C). Results for perennial and permanent wetlands also showed earlier drawdown and/or reduced water levels in future climates (Figs 8C, 8D, 8G and 8H, and S6A and S6B). Our projections also revealed variations in the magnitude of changes that were dependent on wetland type and location.

**Fig 9:**
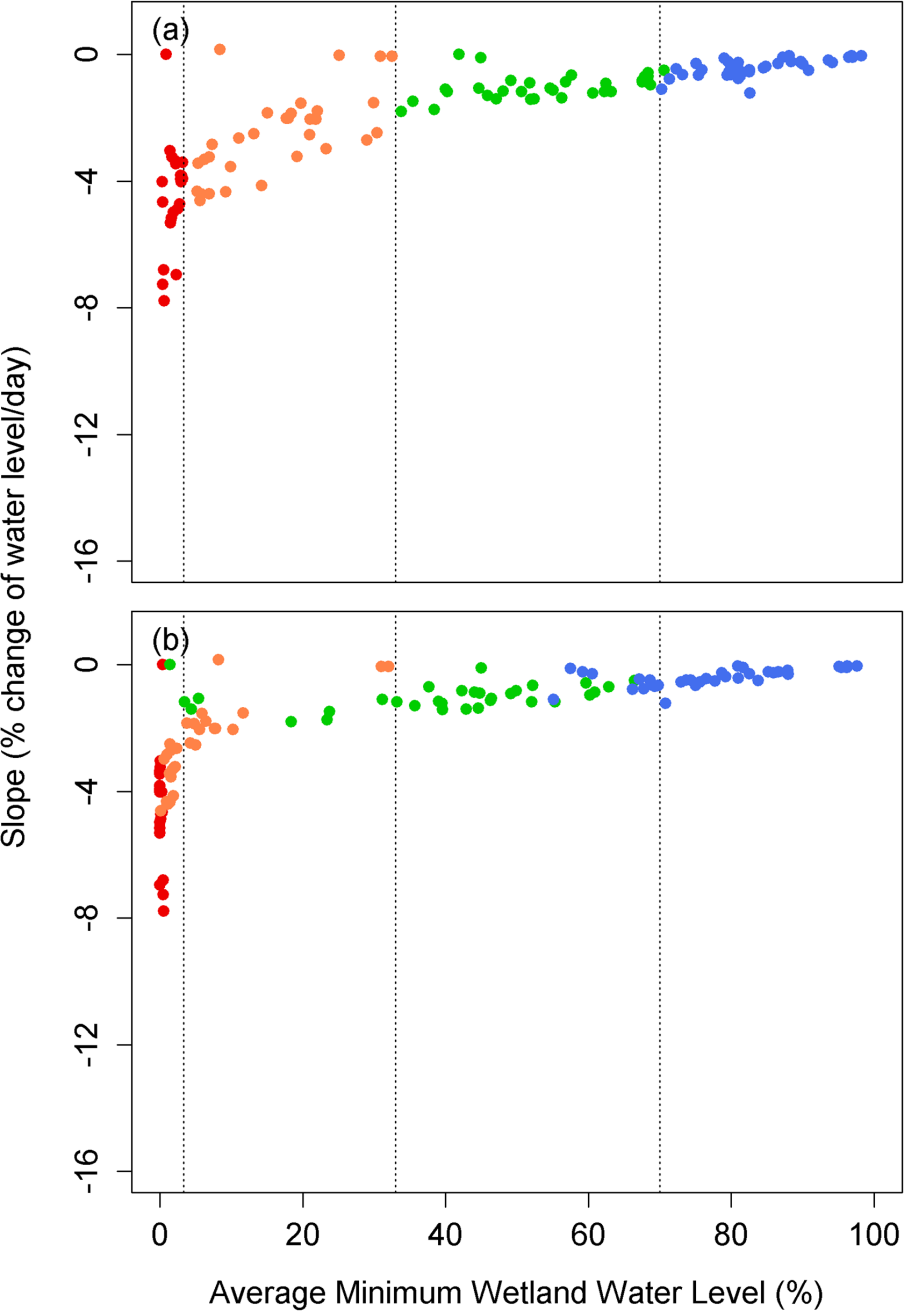
Scatterplots showing the slope of wetland drying versus the average of annual minimum a) for historical runs and b) for the 2080s. Reds are ephemeral hydroperiod wetlands, oranges are intermediate hydroperiod wetland, greens are perennial wetlands and blues are permanent wetlands.

These climate-induced shifts in hydrologic behavior across all types of montane wetlands in our study for which we had site specific models support the hypothesis that climate change will force transitions among wetland types (Fig 9). By the 2080s, 58% of intermediate wetlands are projected to become ephemeral hydroperiod wetlands (17/31 sites), whereas 22% of perennial wetlands are projected to become intermediate wetlands (7/32 sites) and 3% to become ephemeral wetlands (1/32 sites) (Fig 9B). Thirty-two percent of permanent wetlands are projected to become perennial (12/38 sites) (Fig 9B).

**Fig 10:**
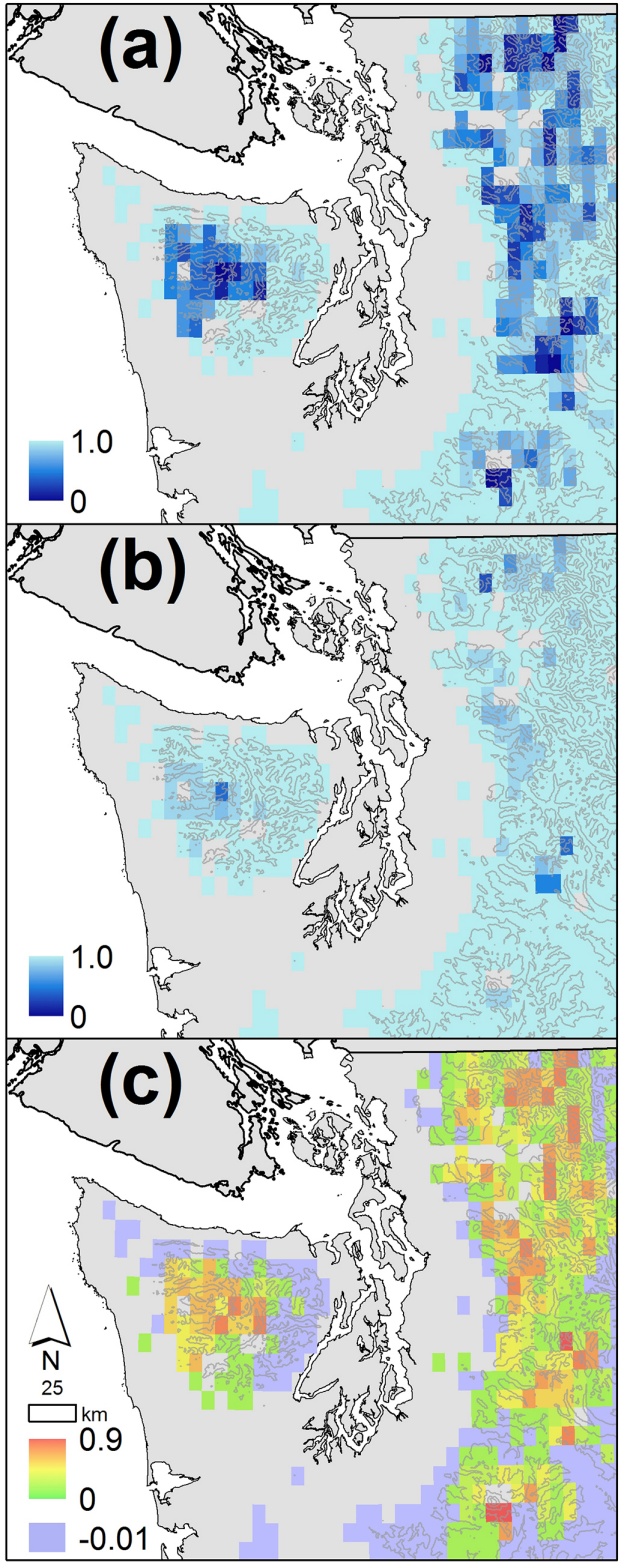
Map of the difference between historical probability of drying and that of the 2080s for intermediate wetlands in the mountains of Western Washington state. Projections for the 2080s are the average value for all ten GCM A1B scenarios. Colored grid cells are above 250m elevation, the region in which our projections are most relevant. Topographic contour intervals are 750m.

Considered at the landscape scale, the projected effects of climate change on future probabilities of drying of intermediate wetlands in mountainous areas of Washington vary geographically but increase overall in higher elevation regions (Fig 10). Lowland areas are typically below the soil moisture threshold every year, and the probability of drying (by this measure) does not change in these areas, thus our model is not appropriate for assessment of lower elevation regions (grey areas on the maps). Similarly, many mid-low elevation regions show little change or even a small shift towards becoming wetter (light green and purple regions on Fig 10C). At higher elevations, both along the crest of the Washington Cascades and in the Olympic Mountains, the probability of drying for intermediate wetlands changes substantially. Under the 20^th^ century climate, many cells had a probability of drying of 0.5 or lower for intermediate wetlands (Fig 10A). By the 2080s, the projected probability of drying is greater than 0.8 for all but a few model cells (Fig 10B). The largest changes in probability of drying were simulated for cells that historically represented the wettest regions in the model domain.

## Discussion

In general, the regression models produced for this study successfully capture historical wetland dynamics during the ecologically important summer drawdown seasons for four different wetland types. However, the regression models were not as robust in capturing different behavior in different years, highlighting the need for longer (>5 year) observed datasets. Additionally, datasets with higher within-year resolution would strengthen the regression approach and enable us to better understand both sources of model error (e.g. geology and soil types, topography, distance from weather stations, etc.) as well as the uncertainty in the range of potential climate impacts and the hydrologic drivers that underlie impacts on wetlands.

Nonetheless, these models are an important first step in understanding wetland response to climate, and strongly suggest that, looking forward, climate change will significantly alter water availability for individual wetlands, which will lead to substantial future shifts in the distribution and composition of wetlands across montane landscapes of the Pacific Northwest. These hydrologic shifts imply widespread changes in the many ecological roles served by wetlands, including: habitat for plants and animals, water storage, groundwater recharge, water quality (filtration), and shifts in nutrient cycling. For wetland-reliant species like amphibians and invertebrates that are particularly sensitive to the timing of water availability in montane areas, the projected changes in wetland hydrologic response are likely to reduce habitat availability and recruitment, and cause declines or extinctions in some regions [28–29].

### Historical model performance

Soil moisture in the bottom soil layer was the best predictor of wetland drawdown for most of the sites investigated, supporting the hypothesis that wetland drawdown cycles are frequently associated with relatively slow drainage and evapotranspiration processes. The regression models demonstrated generally good performance across all four types of montane wetlands within years, successfully capturing the general time series behavior of each wetland. However, regression models performed better in some years than others and simulations were not able to capture all observed variability (e.g. overestimated wetland minimum water level during 2006 in the Deschutes National Forest site, the error in drawdown timing in the Trinity-Alps in 2003, and the missed drawdown timing and/or minimum water levels in Olympic National Park in 2012). These spatial and temporal variations in model performance are expected, and are seen in other comparisons between simulated and observed data at fine spatial scales, such as snow water equivalent measured at individual snow courses [15]. One reason for the discrepancy is that local precipitation is often imperfectly captured in the driving data sets for the VIC model at high elevations (e.g. data from meteorological stations at lower elevations are interpolated to higher elevations in landscapes with high orographic variation). The estimated uncertainty using multiple years of observations in the Oregon and California sites confirmed that our approach is fairly robust in some regions and/or years. However, at times uncertainties are broad enough to be biologically meaningful (e.g. in terms of evaluating risk to particular species). Likewise, the failure of the regression models to effectively capture strongly different patterns of wetland response across very different weather years in Olympic National Park shows that this approach is not universally robust. Additional water-level data would help better evaluate the performance of the models, and likely also improve their performance by allowing additional explanatory variables to be included in the regression models.

### Climate impacts on wetlands

Our climate-change projections demonstrate that all four of the wetland types on which we focused (ephemeral, intermediate, perennial, and permanent wetlands) are likely to experience hydrologic changes in response to future climate. However, the intensity and duration of climate change effects will differ markedly among the four types. These changes are also likely to lead to transitions along the continuum of wetland types captured in our hydrologic classes. Specifically, some ephemeral wetlands may essentially disappear and more than half of currently ecologically productive intermediate montane wetlands are projected to become ephemeral wetlands by the 2080s, as more rapid recession rate and earlier drawdown cause wetlands to reach their bottom volume earlier, resulting in more frequent and longer dry seasons in summer. For some perennial wetlands (e.g. Washington sites), transitions from perennial to intermediate wetlands or even to ephemeral wetlands are also projected as wetland water levels drop under climate change. Driving these changes is the fact that most montane wetlands are located either in snow-dominated watersheds or mixed-rain-and-snow watersheds where snowmelt is a key water source in late spring and summer. Because a warmer climate is likely to cause less snow accumulation in winter and earlier snowmelt in spring, montane wetlands are particularly susceptible to climate change, especially in combination with projected drier summers [17–18].

Comparison between wetlands in Washington and those in Oregon and California also shows that wetlands with seemingly similar hydrologic characteristics are likely to have different sensitivity to climate change depending on local conditions. For example, perennial wetlands in our focal sites in Willamette and Deschutes National Forests, Oregon are likely to be less sensitive to climate change in terms of minimum water level compared to those in Washington. For perennial wetlands in Oregon, simulated summer soil moisture in the VIC simulations was close to residual values nearly every year but the wetlands did not dry out (maintaining ~20% of their maximum depth) under the current climate. This behavior suggests that these wetlands are coupled to more extensive deep groundwater sources, not captured in the VIC simulations, than wetlands in Washington. For the climate-change scenarios, wetlands in Oregon and California are projected to have earlier drawdown and reach their minimum water level earlier, but without drying out. This supports the argument that wetlands connected to deeper groundwater sources are less vulnerable to increased frequency of drying when compared to surface water-fed wetlands [47]. However, because the VIC model does not include a deep groundwater component, more sophisticated modeling approaches (such as the use of fine scale groundwater models) may be required to fully capture these effects [48–49].

### Ecological implications of future wetland change for wetland biota

The broad range of ecological roles played by wetlands means that altered hydrology across whole landscapes will reverberate in many ways, ranging from shifts in wildlife habitat to water storage to patterns of nutrient transfer and transformation. Patterns of soil inundation, for example, determine rates of carbon sequestration and release, nitrogen transformations, and other nutrient cycles. Likewise, changes in temporal pulses of peak water affect local pond metabolism and primary productivity, the structure of plant communities, and patterns of wildlife connectivity [6]. Montane wetlands serve as critical habitat for a wide variety of species, many of which are adapted and sensitive to particular hydrologic regimes that are projected to shift under future climates.

For wetland-reliant species like amphibians and invertebrates that require minimum durations of water availability to breed and complete aquatic life stages, the projected changes in wetland hydrologic response are likely to reduce the number of sites available for breeding, may affect larval densities, individual correlates of fitness such as size at metamorphosis, and reduce recruitment success [29, 50–51]. While many species will be affected by changes in both the timing and frequency of drying, those that metamorphose within a single summer may be more sensitive to changes in the timing of wetland drying. In contrast, species that require multiple years to complete larval development are likely to be most sensitive to changes in the frequency of drying [28]. In wetlands not at risk of drying entirely, ecological effects may also depend on how thermal conditions in ponds change as the climate warms and water levels drop [28, 33, 52]. Finally, as the distribution of wetland hydroperiod changes, so will the spatial distribution of suitable wetland habitats. This may affect important drivers of population and community dynamics such as population connectivity, synchrony, and rates of recolonization [28, 53]. Increased reproductive failures due to drying would further alter local and regional extinction rates [29, 54].

For amphibians in particular, already known to be in decline in many montane regions, climate impacts are likely to interact with non-climate threats such as disease, pollution, and the presence of introduced fish [28, 55–58]. Amphibians and invertebrates are also important prey for many montane species, so population declines in these assemblages could propagate up food webs, negatively affecting the birds, non-avian reptiles, and mammals that rely on them as prey [59–60]. Overall, species’ exposure will depend on what kinds of wetland habitat they use, the current distribution of wetland types across landscapes, and the degree of change in spatial and temporal hydrologic patterns under future climates [28].

### Next steps

Our approach demonstrates a promising first step in relating simulated macro-scale hydrologic variables to observed wetland hydrologic behavior, and projecting the impacts of climate variability and change on montane wetlands in Washington, Oregon, and California. Our relatively robust results across different wetland types, especially given the limited data on which to build regression models, contradict the assumption that wetland dynamics are by definition too complex to model. Challenges remain, however. Because our methods were applied mostly in wetlands with at most a few years of observed hydrologic data (often only a single year), extending these approaches to confirm the robustness of our approach over different ecoregions, additional wetland types, and across a longer time series are essential next steps. These models also need to be coupled to better on-the-ground or remote sensing-derived data on wetland occurrence and type so that the most useful predictions about historical and/or future wetland changes can be made (e.g. existing wetland maps for Mount Rainier National Park miss >50% of the small, biologically productive wetlands that we discuss here) [61]. New remote-sensing methods also make it possible in some cases to reconstruct the historical hydrologic dynamics of wetlands [62], and these in turn provide important validation datasets for retrospective modeling studies. Pairing improved maps with VIC hydrologic projections across large spatial scales will make it possible for the first time to identify hotspots of climate risk to wetlands, how these may interact with other stressors [28]. Overall, however, the models presented here offer a first step in beginning to fill the gap in scientific resources for wetland conservation, vulnerability assessment, and climate adaptation planning at both local and regional scales.

## Supporting Information

S1 Fig Correlation coefficients between observed water level and simulated water balance variables such as precipitation (precip), evapotranspiration (evapot), runoff, soil moistures in the top (soilm1), middle (soilm2) and bottom (soilm3) layers, and snow water equivalent (swe). (TIF)

S2 Fig Correlation coefficients between observed water level and middle (top panel) and bottom soil moisture layers (bottom panel) depending on wetland types, regions, and years when observed data are available. (TIF)

S3 Fig Six wetlands whose goodness of fit (R^2^) between observed and simulated data are less than 0.6. Solid circles show observed data that are used for developing regression model and calculating R^2^ values and open circles are remaining observed data. Blue lines are simulated wetland levels.
(TIF)

S4 Fig Relationship between percentage field capacity and wetland drying for intermediate wetlands across study regions. Spray Park, and Palisades are in Mount Rainier National Park. (There were no intermediate wetlands by our definition in Mazama Ridge.) Deer Lake, Potholes, Clear Lake, and Upper Lena are in Olympic National Park.
(TIF)

S5 Fig Observed wetland water level for year 2000 (a-c) and for year 2012 (d-f) for Olympic National Park compared with. the simulated wetland water levels using the regression models that were developed based on 2000 observation. Solid circles show observed data that are used for developing regression model and/or for calculating R^2^ values and open circles are the other observed data. RMSE is the root mean squared error.
(TIF)

S6 Fig Projected wetland response to climate change for a) Deschutes National Forest, OR, b) Willamette National Forest, OR, and c) Trinity Alps Wilderness, CA. Blue solid lines are wetland hydrographs for years 2003–2004 and red dashed lines show wetland hydrographs of years 2003–2004 with climate change perturbation for the 2080s.
(TIF)

S1 Table List of wetlands used in analysis. Under site locations, MRNP is Mount Rainier National Park, NCNP is North Cascades National Park, and OLYM is Olympic National Park. *Our classifications are based on the long-term dynamics of wetlands from the VIC runs. As a result, some of our classifications at Mazama Ridge differ from Girdner and Larson’s classification from the very dry summer of 1992.
(DOCX)

S2 Table List of parameters used for regression models shown in Figs 5–7 and S4. (DOCX)
