# Correction: Projecting the Hydrologic Impacts of Climate Change on Montane Wetlands

**DOI:** 10.1371/journal.pone.0142960

**Published:** 2015-11-10

**Authors:** Se-Yeun Lee, Maureen E. Ryan, Alan F. Hamlet, Wendy J. Palen, Joshua J. Lawler, Meghan Halabisky

The caption for Fig 10 is incorrect. Please see Fig 10 and the corrected caption here.

**Fig 10:**
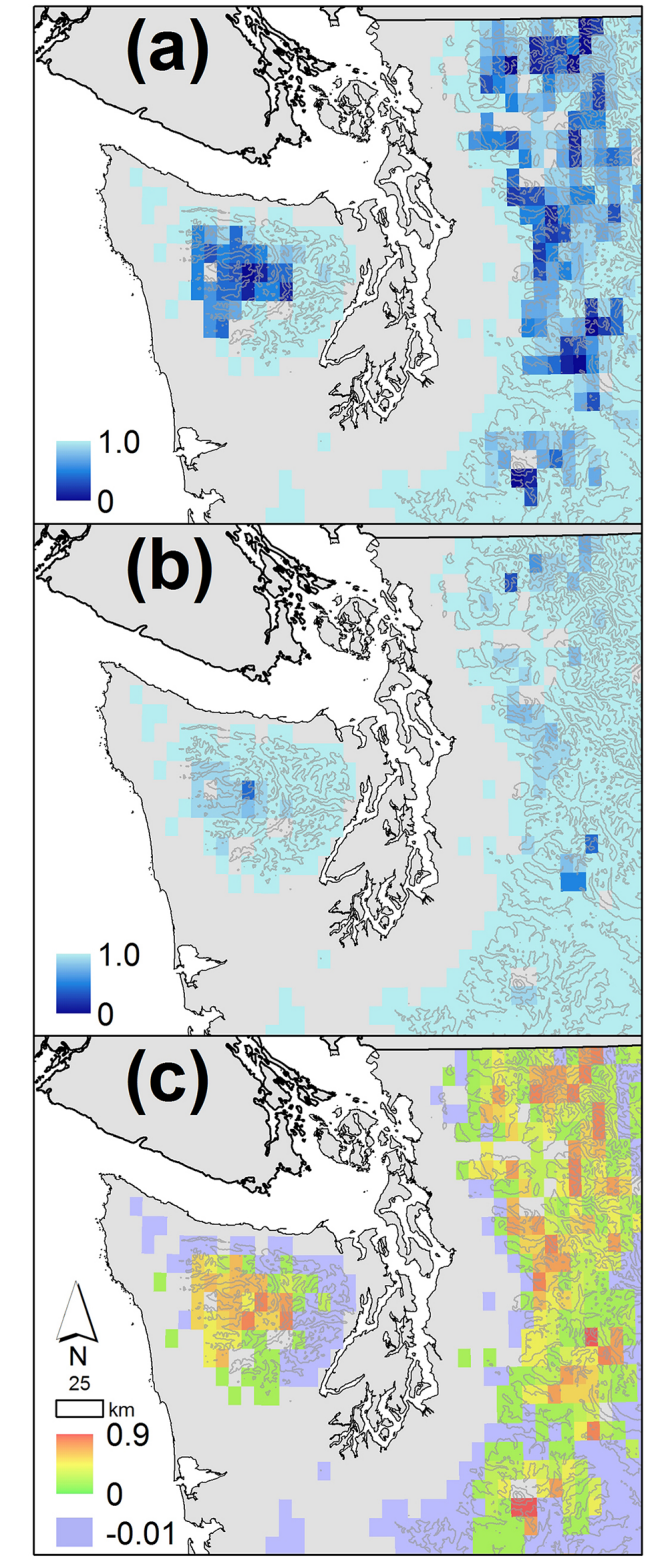
Maps of the changing probability of drying for intermediate wetlands in the mountains of Western Washington state. (a) the probability of drying for historical runs, (b) the probability of drying for the 2080s, and (c) the difference between historical probability of drying and that of the 2080s for intermediate wetlands. Projections for the 2080s are the average value for all ten GCM A1B scenarios. Colored grid cells are above 250m elevation, the region in which our projections are most relevant. Topographic contour intervals are 750m.
