# Supplementary figures and images for: Projecting the Hydrologic Impacts of Climate Change on Montane Wetlands
Source: PLoS One. 2015 Sep 2;10(9):e0136385. doi: 10.1371/journal.pone.0136385 (PMC4557981; doi:10.1371/journal.pone.0136385)

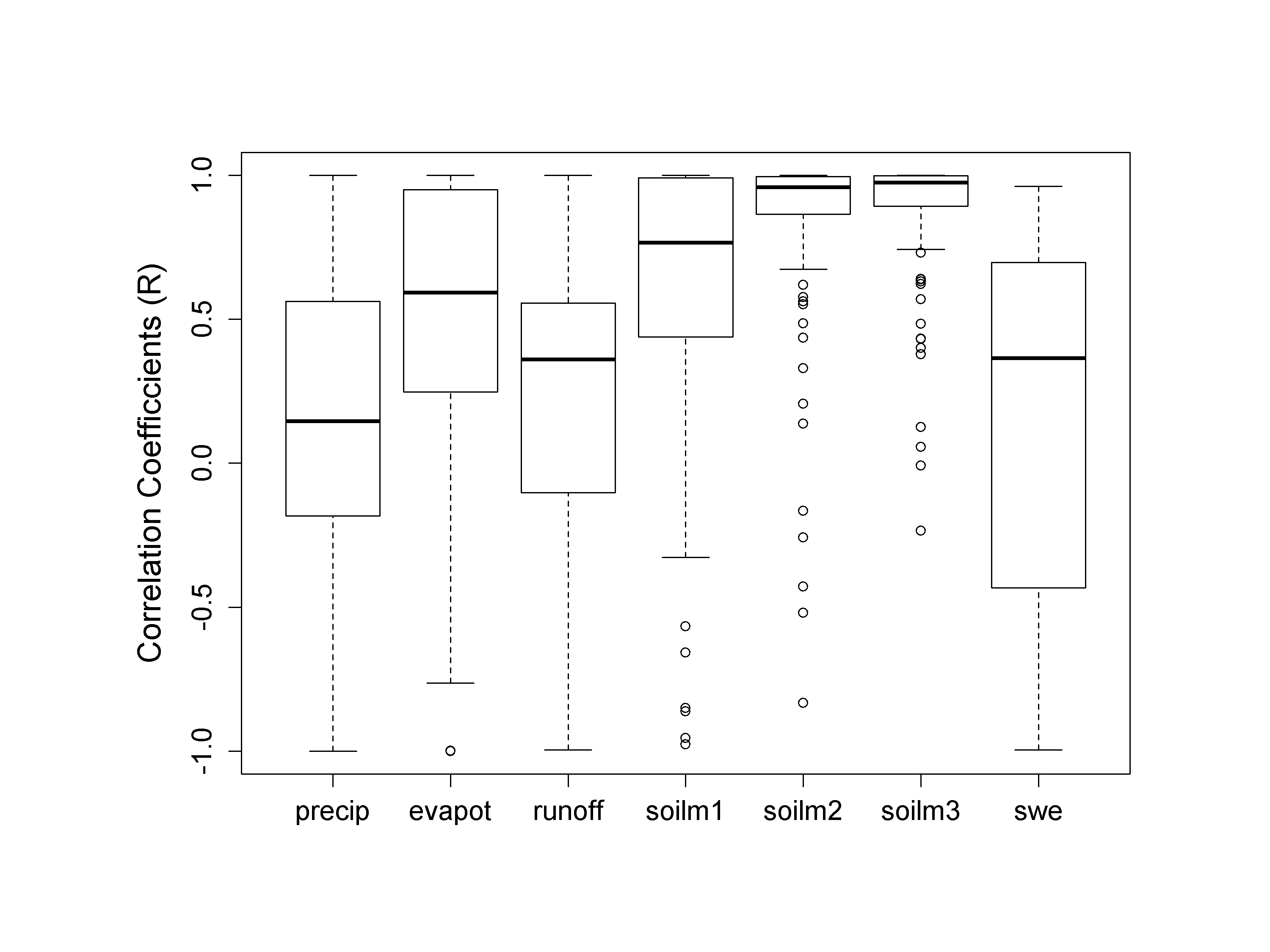

Supplement: S1 Fig — (TIF) [file pone.0136385.s001.tif]

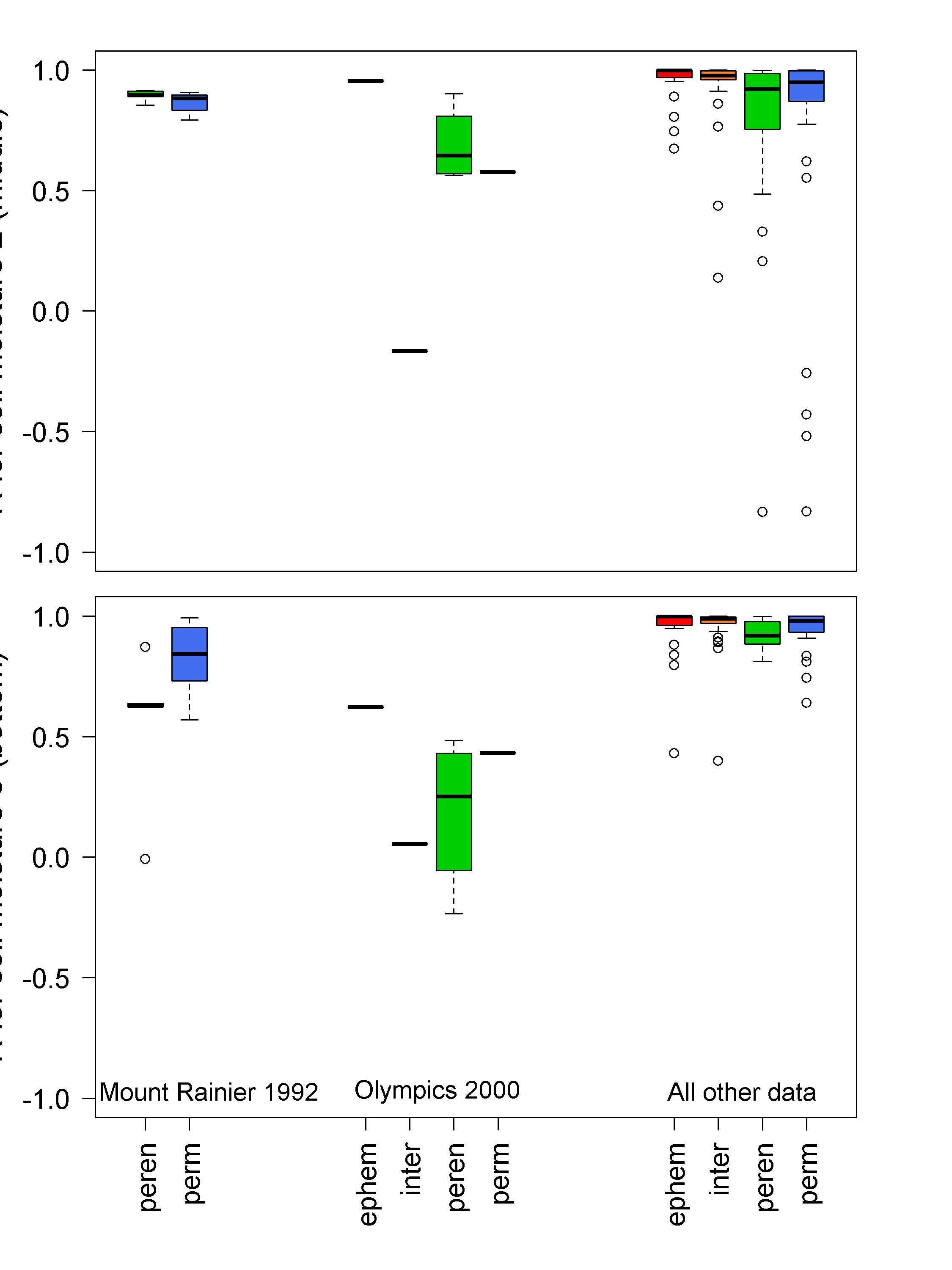

Supplement: S2 Fig — (TIF) [file pone.0136385.s002.tif]

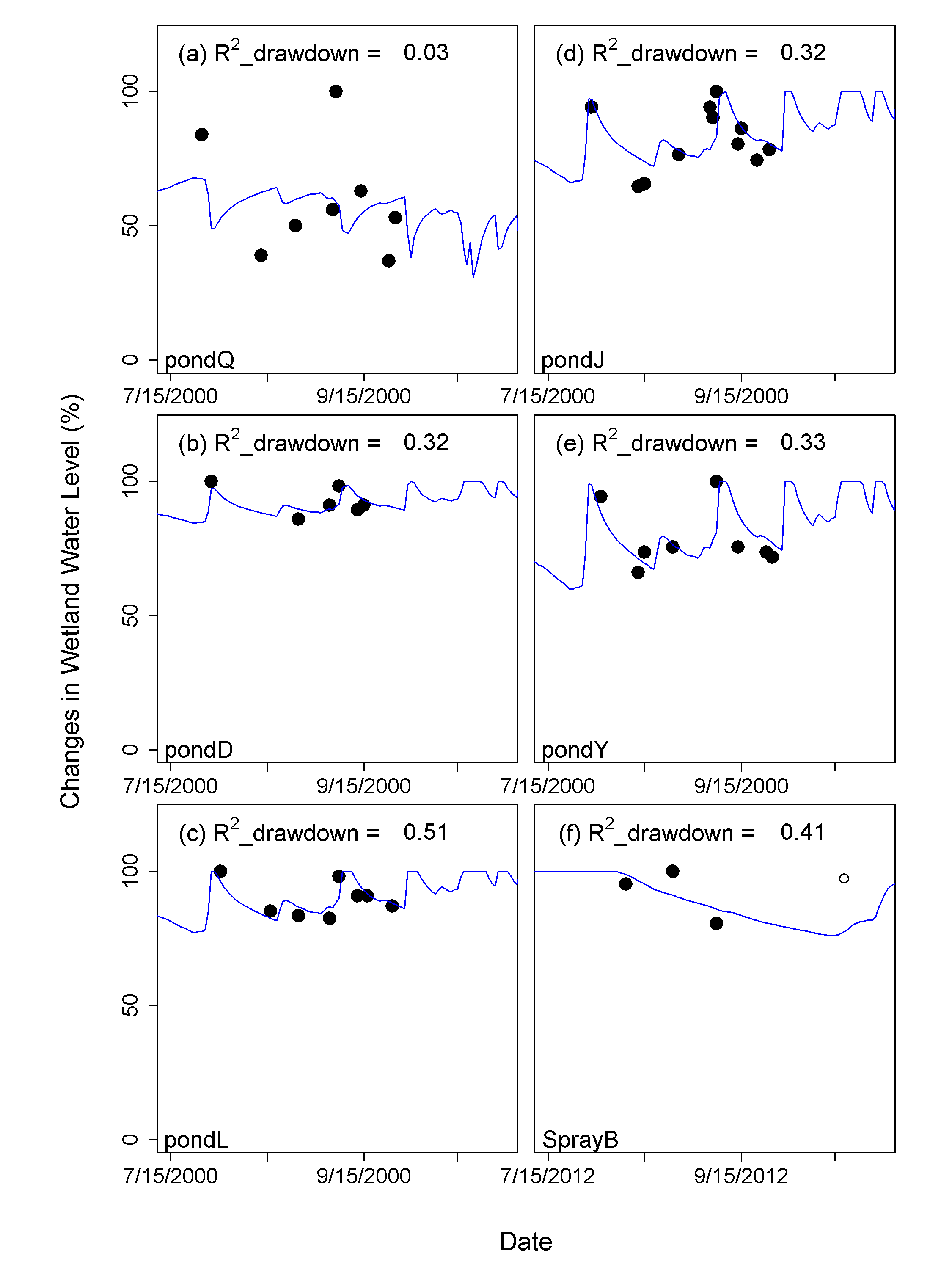

Supplement: S3 Fig — Solid circles show observed data that are used for developing regression model and calculating R2 values and open circles are remaining observed data. Blue lines are simulated wetland levels. (TIF) [file pone.0136385.s003.tif]

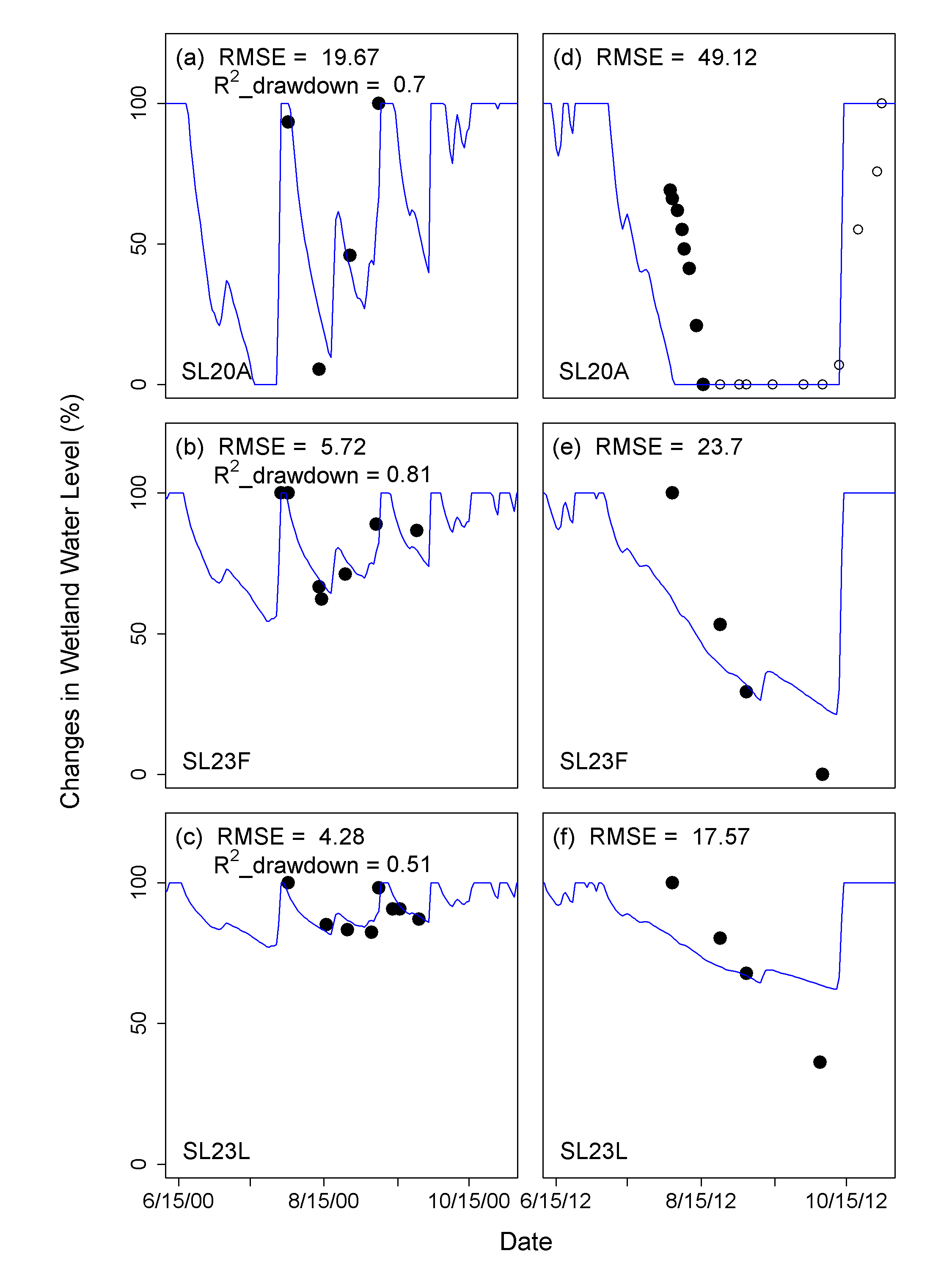

Supplement: S4 Fig — Spray Park, and Palisades are in Mount Rainier National Park. (There were no intermediate wetlands by our definition in Mazama Ridge.) Deer Lake, Potholes, Clear Lake, and Upper Lena are in Olympic National Park. (TIF) [file pone.0136385.s004.tif]

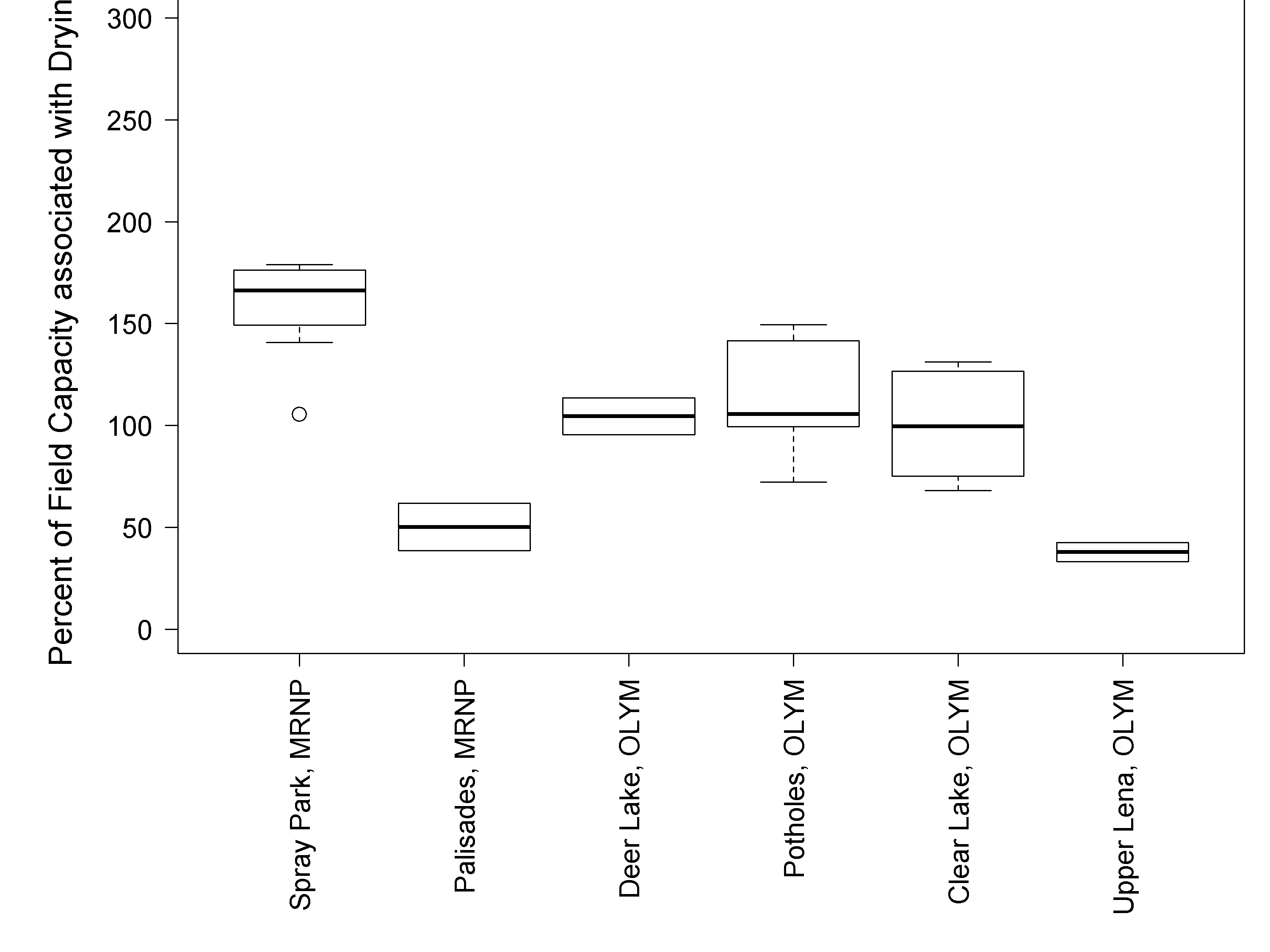

Supplement: S5 Fig — Solid circles show observed data that are used for developing regression model and/or for calculating R2 values and open circles are the other observed data. RMSE is the root mean squared error. (TIF) [file pone.0136385.s005.tif]

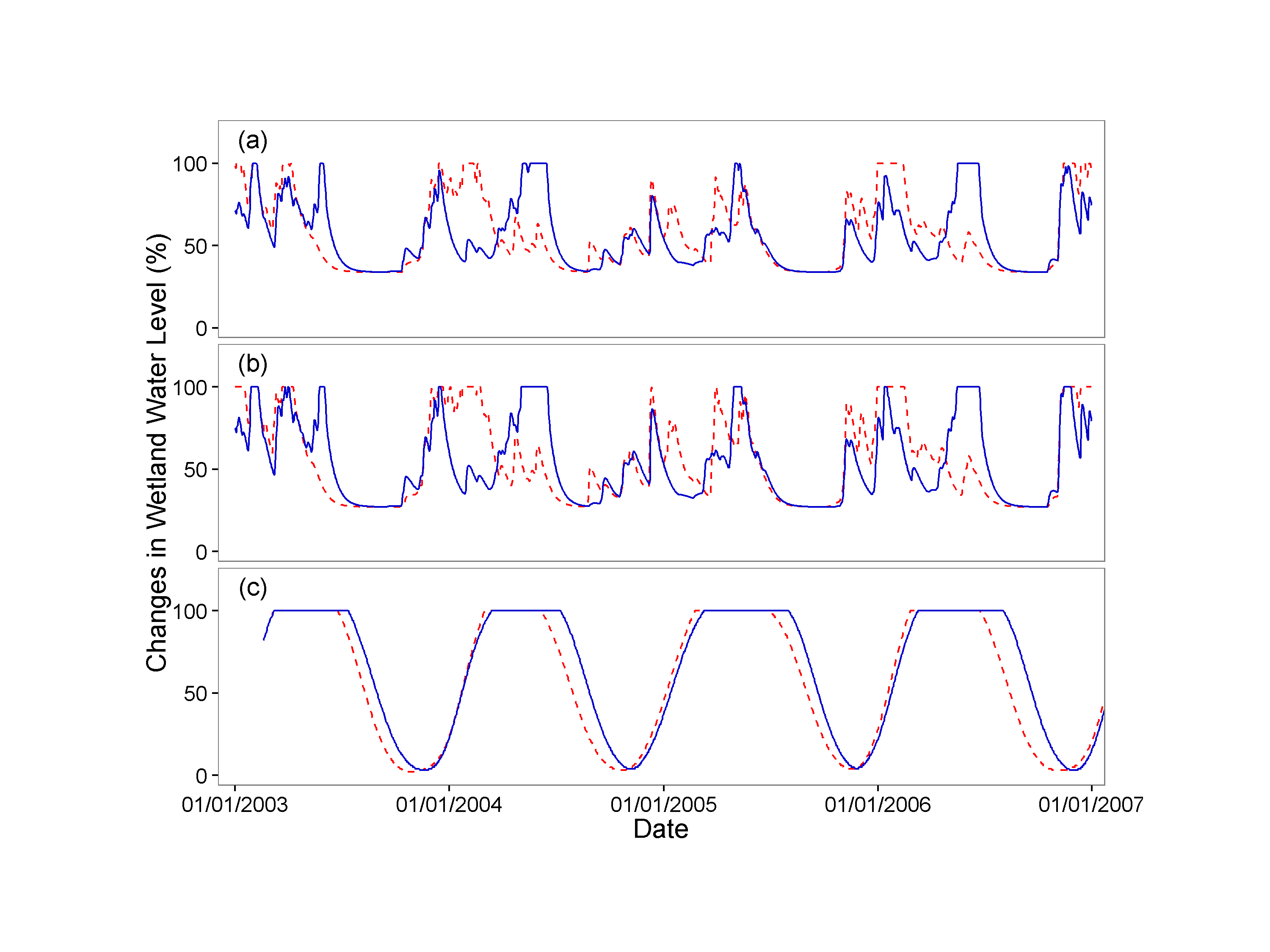

Supplement: S6 Fig — Blue solid lines are wetland hydrographs for years 2003–2004 and red dashed lines show wetland hydrographs of years 2003–2004 with climate change perturbation for the 2080s. (TIF) [file pone.0136385.s006.tif]
