# Supplementary material for: Projecting the Hydrologic Impacts of Climate Change on Montane Wetlands
Source: PLoS One. 2015 Sep 2;10(9):e0136385. doi: 10.1371/journal.pone.0136385 (PMC4557981; doi:10.1371/journal.pone.0136385)
Supplement: S1 Table — Under site locations, MRNP is Mount Rainier National Park, NCNP is North Cascades National Park, and OLYM is Olympic National Park. *Our classifications are based on the long-term dynamics of wetlands from the VIC runs. As a result, some of our classifications at Mazama Ridge differ from Girdner and Larson’s classification from the very dry summer of 1992. (DOCX) [file pone.0136385.s007.docx]

**Table S1.** List of wetlands used in analysis. Under site locations, MRNP is Mount Rainier National Park, NCNP is North Cascades National Park, and OLYM is Olympic National Park. *Our classifications are based on the long-term dynamics of wetlands from the VIC runs. As a result, some of our classifications at Mazama Ridge differ from Girdner and Larson’s classification from the very dry summer of 1992.

| Site Location | Type | Name | Method | Observation Years |
| --- | --- | --- | --- | --- |
| Mazama Ridge, MRNP, WA | Perennial | Far 2 (Noname)* | measurement | 2 years (1992, 2012) |
| Mazama Ridge, MRNP, WA | Perennial | High A (LZ16)* | measurement | 2 years (1992, 2012) |
| Mazama Ridge, MRNP, WA | Perennial | High G (LZ18)* | measurement | 2 years (1992, 2012) |
| Mazama Ridge, MRNP, WA | Perennial | High D (LZ14) | measurement, iButton | 2 years (1992, 2012) |
| Mazama Ridge, MRNP, WA | Permanent | High B (M16)* | measurement | 2 years (1992, 2012) |
| Mazama Ridge, MRNP, WA | Permanent | High C (LZ15) | measurement | 2 years (1992, 2012) |
| Mazama Ridge, MRNP, WA | Permanent | Far 1 (LZ19) | measurement | 2 years (1992, 2012) |
| Mazama Ridge, MRNP, WA | Permanent | Far 3 (LZ17) | measurement, iButton | 2 years (1992, 2012) |
| Mazama Ridge, MRNP, WA | Perennial | M10 | measurement | 1 year (1992) |
| Mazama Ridge, MRNP, WA | Permanent | LZ12 | measurement | 1 year (1992) |
| Palisades, MRNP, WA | Permanent | Pal1 | measurement | 1 year (2012) |
| Palisades, MRNP, WA | Permanent | Pal2 | measurement | 1 year (2012) |
| Palisades, MRNP, WA | Ephemeral | Pal3 | iButton | 1 year (2012) |
| Palisades, MRNP, WA | Intermediate | Pal5 | measurement | 1 year (2012) |
| Palisades, MRNP, WA | Perennial | Pal6 | measurement | 1 year (2012) |
| Palisades, MRNP, WA | Permanent | Pal8 | measurement | 1 year (2012) |
| Palisades, MRNP, WA | Intermediate | Pal9 | measurement | 1 year (2012) |
| Palisades, MRNP, WA | Permanent | Pal10 | measurement | 1 year (2012) |
| Spray Park, MRNP, WA | Permanent | SprayA | measurement | 1 year (2012) |
| Spray Park, MRNP, WA | Permanent | SprayB | measurement | 1 year (2012) |
| Spray Park, MRNP, WA | Perennial | SprayC | iButton | 1 year (2012) |
| Spray Park, MRNP, WA | Intermediate | SprayD | measurement | 1 year (2012) |
| Spray Park, MRNP, WA | Ephemeral | SprayE | iButton | 1 year (2012) |
| Spray Park, MRNP, WA | Permanent | SprayF | measurement | 1 year (2012) |
| Spray Park, MRNP, WA | Ephemeral | SprayG | measurement | 1 year (2012) |
| Spray Park, MRNP, WA | Intermediate | SprayH | measurement | 1 year (2012) |
| Spray Park, MRNP, WA | Intermediate | SprayI | measurement | 1 year (2012) |
| Spray Park, MRNP, WA | Permanent | SprayJ1 | measurement | 1 year (2012) |
| Spray Park, MRNP, WA | Intermediate | SprayJ2 | measurement | 1 year (2012) |
| Spray Park, MRNP, WA | Intermediate | SprayK | measurement | 1 year (2012) |
| Spray Park, MRNP, WA | Ephemeral | SprayK.A1 | measurement | 1 year (2012) |
| Spray Park, MRNP, WA | Ephemeral | SprayK.A2 | measurement | 1 year (2012) |
| Spray Park, MRNP, WA | Intermediate | SprayK.A3 | measurement | 1 year (2012) |
| Spray Park, MRNP, WA | Intermediate | SprayL | measurement | 1 year (2012) |
| Spray Park, MRNP, WA | Intermediate | SprayM | measurement | 1 year (2012) |
| Spray Park, MRNP, WA | Ephemeral | SprayN | measurement | 1 year (2012) |
| Spray Park, MRNP, WA | Permanent | SprayO | measurement | 1 year (2012) |
| Spray Park, MRNP, WA | Permanent | SprayQ | measurement | 1 year (2012) |
| NCNP, WA | Permanent | Pyramid | iButton | 1 year (2012) |
| NCNP, WA | Permanent | Thunder | measurement | 1 year (2012) |
| Deer Lake, OLYM, WA | Perennial | Deer Camp1 | measurement | 1 year (2012) |
| Deer Lake, OLYM, WA | Permanent | Deer Camp2 | measurement | 1 year (2012) |
| Deer Lake, OLYM, WA | Ephemeral | Deer Camp3 | measurement | 1 year (2012) |
| Deer Lake, OLYM, WA | Permanent | Deer Camp4 | measurement | 1 year (2012) |
| Deer Lake, OLYM, WA | Perennial | Deer Trail1 | measurement | 1 year (2012) |
| Deer Lake, OLYM, WA | Intermediate | Deer Trail2 | measurement | 1 year (2012) |
| Deer Lake, OLYM, WA | Permanent | Deer Meadow1.2 | measurement | 1 year (2012) |
| Deer Lake, OLYM, WA | Perennial | Deer Meadow3 | measurement | 1 year (2012) |
| Deer Lake, OLYM, WA | Ephemeral | Deer Meadow4S | measurement | 1 year (2012) |
| Deer Lake, OLYM, WA | Ephemeral | Deer Meadow5 | measurement | 1 year (2012) |
| Deer Lake, OLYM, WA | Permanent | Deer Meadow6 | measurement | 1 year (2012) |
| Deer Lake, OLYM, WA | Ephemeral | Deer Meadow7 | measurement | 1 year (2012) |
| Deer Lake, OLYM, WA | Ephemeral | Deer Meadow8 | measurement | 1 year (2012) |
| Deer Lake, OLYM, WA | Perennial | Deer Meadow9 | iButton | 1 year (2012) |
| Deer Lake, OLYM, WA | Intermediate | Deer Meadow10 | measurement | 1 year (2012) |
| Deer Lake, OLYM, WA | Permanent | Deer Meadow12 | measurement | 1 year (2012) |
| Potholes, OLYM, WA | Intermediate | PM1a | measurement | 1 year (2012) |
| Potholes, OLYM, WA | Intermediate | PM2a | measurement | 1 year (2012) |
| Potholes, OLYM, WA | Intermediate | PM3a | measurement | 1 year (2012) |
| Potholes, OLYM, WA | Permanent | PM3b | measurement | 1 year (2012) |
| Potholes, OLYM, WA | Intermediate | PM4a | measurement | 1 year (2012) |
| Potholes, OLYM, WA | Ephemeral | PM5a | measurement | 1 year (2012) |
| Potholes, OLYM, WA | Intermediate | PM6a | measurement | 1 year (2012) |
| Potholes, OLYM, WA | Ephemeral | PM6c | measurement | 1 year (2012) |
| Potholes, OLYM, WA | Permanent | PotholeA | measurement | 1 year (2012) |
| Potholes, OLYM, WA | Ephemeral | PotholeB | measurement | 1 year (2012) |
| Potholes, OLYM, WA | Intermediate | PotholeC | measurement | 1 year (2012) |
| Potholes, OLYM, WA | Permanent | PotholeD | measurement | 1 year (2012) |
| Potholes, OLYM, WA | Permanent | PotholeE | measurement | 1 year (2012) |
| Potholes, OLYM, WA | Intermediate | PotholeF | measurement | 1 year (2012) |
| Potholes, OLYM, WA | Permanent | PotholeG | measurement | 1 year (2012) |
| Potholes, OLYM, WA | Ephemeral | PotholeH | measurement | 1 year (2012) |
| Upper Lena, OLYM, WA | Permanent | MUL2 | measurement | 1 year (2012) |
| Upper Lena, OLYM, WA | Perennial | MUL2a | measurement | 1 year (2012) |
| Upper Lena, OLYM, WA | Perennial | MUL3 | measurement | 1 year (2012) |
| Upper Lena, OLYM, WA | Perennial | MUL3a | measurement | 1 year (2012) |
| Upper Lena, OLYM, WA | Permanent | MUL3b | measurement | 1 year (2012) |
| Upper Lena, OLYM, WA | Ephemeral | MUL4 | measurement | 1 year (2012) |
| Upper Lena, OLYM, WA | Perennial | MUL7a | measurement | 1 year (2012) |
| Upper Lena, OLYM, WA | Perennial | MUL8 | measurement | 1 year (2012) |
| Upper Lena, OLYM, WA | Permanent | MUL9 | measurement | 1 year (2012) |
| Upper Lena, OLYM, WA | Perennial | MUL10 | measurement | 1 year (2012) |
| Upper Lena, OLYM, WA | Permanent | MUL11 | measurement | 1 year (2012) |
| Upper Lena, OLYM, WA | Ephemeral | MUL11a | measurement | 1 year (2012) |
| Upper Lena, OLYM, WA | Permanent | MUL11b | measurement | 1 year (2012) |
| Upper Lena, OLYM, WA | Permanent | MUL11c | measurement | 1 year (2012) |
| Upper Lena, OLYM, WA | Permanent | MUL11d | measurement | 1 year (2012) |
| Upper Lena, OLYM, WA | Intermediate | MUL11e | measurement | 1 year (2012) |
| Upper Lena, OLYM, WA | Permanent | MUL11f | measurement | 1 year (2012) |
| Upper Lena, OLYM, WA | Perennial | MUL11g | measurement | 1 year (2012) |
| Upper Lena, OLYM, WA | Ephemeral | MUL12a | measurement | 1 year (2012) |
| Upper Lena, OLYM, WA | Perennial | MUL12b | measurement | 1 year (2012) |
| Upper Lena, OLYM, WA | Intermediate | MUL13b | measurement | 1 year (2012) |
| Clear Lake, OLYM, WA | Perennial | SL20 | measurement | 1 year (2012) |
| Clear Lake, OLYM, WA | Ephemeral | SL20A (Pond V) | measurement, iButton | 2 years (2000, 2012) |
| Clear Lake, OLYM, WA | Perennial | SL20D | measurement | 1 year (2012) |
| Clear Lake, OLYM, WA | Intermediate | SL20E | measurement | 1 year (2012) |
| Clear Lake, OLYM, WA | Permanent | SL20F | measurement | 1 year (2012) |
| Clear Lake, OLYM, WA | Permanent | SL20H | measurement | 1 year (2012) |
| Clear Lake, OLYM, WA | Perennial | SL20I (Pond J) | measurement | 2 years (2000, 2012) |
| Clear Lake, OLYM, WA | Permanent | SL23A (Pond D) | measurement | 2 years (2000, 2012) |
| Clear Lake, OLYM, WA | Perennial | SL23B | measurement | 1 year (2012) |
| Clear Lake, OLYM, WA | Perennial | SL23C (Pond Y) | measurement | 2 years (2000, 2012) |
| Clear Lake, OLYM, WA | Intermediate | SL23D-M | measurement | 1 year (2012) |
| Clear Lake, OLYM, WA | Intermediate | SL23D-N | measurement | 1 year (2012) |
| Clear Lake, OLYM, WA | Ephemeral | SL23E | measurement | 1 year (2012) |
| Clear Lake, OLYM, WA | Perennial | SL23F (Pond K) | measurement | 2 years (2000, 2012) |
| Clear Lake, OLYM, WA | Ephemeral | SL23G | measurement | 1 year (2012) |
| Clear Lake, OLYM, WA | Intermediate | SL23H (Pond Q) | measurement | 1 year (2000, 2012) |
| Clear Lake, OLYM, WA | Perennial | SL23I | iButton | 1 year (2012) |
| Clear Lake, OLYM, WA | Ephemeral | SL23J | iButton | 1 year (2012) |
| Clear Lake, OLYM, WA | Perennial | SL23K | iButton | 1 year (2012) |
| Clear Lake, OLYM, WA | Perennial | SL23L (Pond L) | measurement | 2 years (2000, 2012) |
| Clear Lake, OLYM, WA | Perennial | SL26 | measurement | 1 year (2012) |
| Clear Lake, OLYM, WA | Perennial | SL26A | measurement | 1 year (2012) |
| Clear Lake, OLYM, WA | Perennial | SL26B | iButton | 1 year (2012) |
| Clear Lake, OLYM, WA | Intermediate | SL27B | measurement | 1 year (2012) |
| Clear Lake, OLYM, WA | Intermediate | SL27D | measurement | 1 year (2012) |
| Clear Lake, OLYM, WA | Intermediate | SL27E.1 | measurement | 1 year (2012) |
| Clear Lake, OLYM, WA | Ephemeral | SL27E.2 | measurement | 1 year (2012) |
| Clear Lake, OLYM, WA | Ephemeral | SL.3extras | measurement | 1 year (2012) |
| Deschutes NF, OR | Perennial | Muskrat | measurement | 2 years (2003, 2006) |
| Willamette NF, OR | Perennial | Penn | measurement | 3 years (2004-2006) |
| Willamette NF, OR | Intermediate | Unnamed | measurement | 3 years (2003, 2005, 2006) |
| Trinity Alps, CA | Intermediate | Snowmelt | measurement | 5 years (2003-2007) |
