# Supplementary material for: Projecting the Hydrologic Impacts of Climate Change on Montane Wetlands
Source: PLoS One. 2015 Sep 2;10(9):e0136385. doi: 10.1371/journal.pone.0136385 (PMC4557981; doi:10.1371/journal.pone.0136385)
Supplement: S2 Table — (DOCX) [file pone.0136385.s008.docx]

**Table S2.** List of parameters used for regression models shown in Figures 5-7 & S4.

| Name | Slope | Y-Intercept | Predictor | Fluxes File | Figure |
| --- | --- | --- | --- | --- | --- |
| SprayE | 1.9 | -403.3 | Soilm3 | fluxes_46.90625_-121.84375 | Fig.5 |
| Pal5 | 0.6 | -22.3 | Soilm3 | fluxes_46.90625_-121.59375 |  |
| SprayC | 0.3 | 13.9 | Soilm3 | fluxes_46.90625_-121.84375 |  |
| Far 3 (LZ17) | 0.4 | 76.1 | Soilm2 | fluxes_46.78125_-121.71875 |  |
| SL23J | 0.8 | -163.9 | Soilm3 | fluxes_47.90625_-123.78125 |  |
| PM1a | 0.8 | -136.5 | Soilm3 | fluxes_47.90625_-123.84375 |  |
| SL23K | 0.3 | 10.2 | Soilm3 | fluxes_47.90625_-123.78125 |  |
| Deer Camp4 | 0.1 | 80.1 | Soilm3 | fluxes_47.90625_-123.84375 |  |
| Muskrat | 0.4 | 30.8 | Soilm3 | fluxes_43.90625_-121.90625 | Fig.6 |
| Unnamed | 0.3 | 32.4 | Soilm3 | fluxes_43.90625_-121.90625 |  |
| Snowmelt | 0.3 | 37.5 | Soilm3 | fluxes_40.90625_-122.90625 |  |
| Far 2 (Noname) | 3.9 | -136.6 | Soilm2 | fluxes_46.78125_-121.71875 | Fig.7 |
| High D (LZ14) | 2.0 | -24.5 | Soilm2 | fluxes_46.78125_-121.71875 |  |
| Far 1 (LZ19) | 0.5 | 69.4 | Soilm2 | fluxes_46.78125_-121.71875 |  |
| SL20A (Pond V) | 7.0 | -348.7 | Soilm2 | fluxes_47.90625_-123.78125 | Fig.S4 |
| SL23F (Pond K) | 2.2 | -48.2 | Soilm2 | fluxes_47.90625_-123.78125 |  |
| SL23L (Pond L) | 1.0 | 30.6 | Soilm2 | fluxes_47.90625_-123.78125 |  |
